# Potent neutralizing anti-SARS-CoV-2 human antibodies cure infection with SARS-CoV-2 variants in hamster model

**DOI:** 10.1016/j.isci.2022.104705

**Published:** 2022-07-03

**Authors:** Maya Imbrechts, Wim Maes, Louanne Ampofo, Nathalie Van den Berghe, Bas Calcoen, Dominique Van Looveren, Winnie Kerstens, Madina Rasulova, Thomas Vercruysse, Sam Noppen, Rana Abdelnabi, Caroline Foo, Kevin Hollevoet, Piet Maes, Xin Zhang, Dirk Jochmans, Karen Ven, Jeroen Lammertyn, Karen Vanhoorelbeke, Nico Callewaert, Paul De Munter, Dominique Schols, Hendrik Jan Thibaut, Johan Neyts, Paul Declerck, Nick Geukens

**Affiliations:** 1 KU Leuven, PharmAbs: the KU Leuven Antibody Center, Herestraat 49 box 820, 3000 Leuven, Belgium; 2 KU Leuven, Department Pharmaceutical and Pharmacological Sciences, Laboratory for Therapeutic and Diagnostic Antibodies, 3000 Leuven, Belgium; 3 KU Leuven, MabMine: KU Leuven Single B Cell Mining Platform, Herestraat 49 box 820, 3000 Leuven, Belgium; 4 KU Leuven Campus Kortrijk, IRF Life Sciences, Laboratory for Thrombosis Research, 3000 Leuven, Belgium; 5 KU Leuven, Department of Microbiology, Immunology and Transplantation, Rega Institute for Medical Research, Laboratory of Virology and Chemotherapy, 3000 Leuven, Belgium; 6 Molecular Vaccinology and Vaccine Discovery, 3000 Leuven, Belgium; 7 KU Leuven Department of Microbiology, Immunology and Transplantation, Rega Institute, Translational Platform Virology and Chemotherapy (TPVC), KU Leuven, Leuven, Belgium; 8 GVN, Global Virus Network; 9 KU Leuven, Department of Biosystems, Biosensors Group, 3000 Leuven, Belgium; 10 AZ Groeninge Hospital Clinical Laboratory, 8500 Kortrijk, Belgium; 11 Department of Internal Medicine, University Hospitals Leuven, 3000 Leuven, Belgium; 12 KU Leuven, Department of Microbiology, Immunology and Transplantation, Laboratory for Clinical Infectious and Inflammatory Disorders, 3000 Leuven, Belgium

**Keywords:** Immunology, Virology

## Abstract

Treatment with neutralizing monoclonal antibodies (mAbs) against severe acute respiratory syndrome coronavirus 2 (SARS-CoV-2) contributes to COVID-19 management. Unfortunately, SARS-CoV-2 variants escape several of these recently approved mAbs, highlighting the need for additional discovery and development. In a convalescent patient with COVID-19, we identified six mAbs, classified in four epitope groups, that potently neutralized SARS-CoV-2 D614G, beta, gamma, and delta infection *in vitro*, with three mAbs neutralizing omicron as well. In hamsters, mAbs 3E6 and 3B8 potently cured infection with SARS-CoV-2 Wuhan, beta, and delta when administered post-viral infection at 5 mg/kg. Even at 0.2 mg/kg, 3B8 still reduced viral titers. Intramuscular delivery of DNA-encoded 3B8 resulted in *in vivo* mAb production of median serum levels up to 90 μg/mL, and protected hamsters against delta infection. Overall, our data mark 3B8 as a promising candidate against COVID-19, and highlight advances in both the identification and gene-based delivery of potent human mAbs.

## Highlights

• Discovery of potent neutralizing antibodies classified in different epitope groups
• Antibodies neutralize SARS-CoV-2 D614G, beta, gamma, delta, and omicron *in vitro*
• Selected antibodies potently treat SARS-CoV-2 infection in hamsters at low doses
• Intramuscular delivery of DNA-encoded 3B8 protects hamsters against infection

## Graphical abstract

**Figure:**
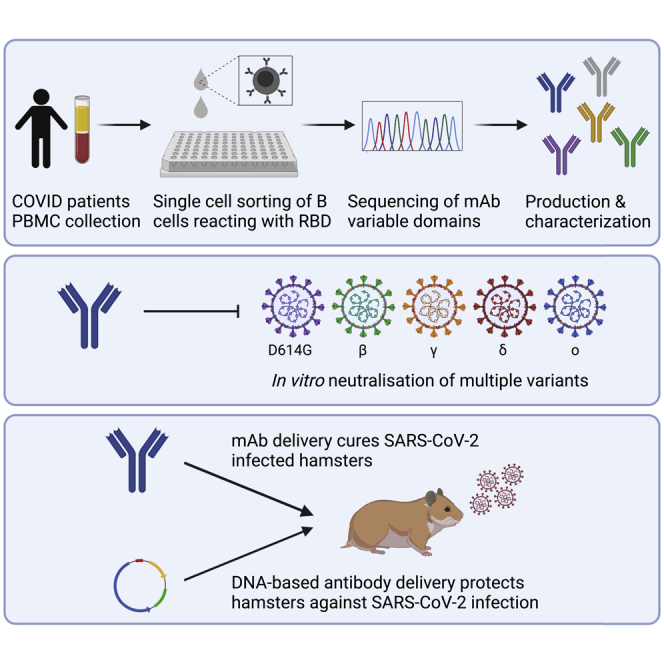

## Introduction

The COVID-19 pandemic, caused by infection with severe acute respiratory syndrome coronavirus 2 (SARS-CoV-2), has resulted in an unprecedented global health and economic crisis and has already caused more than five million deaths worldwide (Ritchie et al., 2021). The spike protein of SARS-CoV-2 consists of an S1 subunit that recognizes host cell receptors and an S2 subunit that promotes membrane fusion of virus and host cells. Within the S1 subunit, the receptor-binding domain (RBD) is responsible for interaction with receptor angiotensin-converting enzyme 2 (ACE2) on host cells to mediate viral entry. Consequently, SARS-CoV-2 spike protein is the major target of neutralizing antibodies (Abs) (Walls et al., 2020; Wrapp et al., 2020).

Antibodies can be elicited by natural infection and vaccination, or can be administered as recombinant monoclonal antibodies (mAbs) in a passive immunization strategy. Although vaccines are essential tools to fight this pandemic, therapeutic modalities, including mAbs, can also play a crucial role. This is especially the case for (immune-compromised or elderly) individuals who may not generate a robust response to their vaccine, cannot be vaccinated, are at high risk for severe illness, or are still awaiting their vaccine (Taylor et al., 2021). Recent advances in mAb discovery, combined with the favorable safety profile and clinical experience, make these ideal molecules for such deployment.

As of February 2022, five human mAb treatments have received (emergency use) authorization: casirivimab/imdevimab REGN-COV2 (i.e. REGN10933 + REGN10987) from Regeneron (Europe), regdanvimab (CT-P59) from Celltrion (Europe), sotrovimab (VIR-7831) from Vir Biotechnology/GlaxoSmithKline (Europe and US), bebtelovimab (Ly-CoV1404) from AbCellera & Eli Lilly (US), and tixagevimab/cilgavimab from AstraZeneca (US, approved for pre-exposure prophylaxis only) (*US Food and Drug Administration: COVID-19 EUA information*, 2022; European Medicines Agency, 2022*: COVID-19 Treatments*, 2022).

Unfortunately, SARS-CoV-2 variants beta, gamma, delta, and omicron escape from some of the currently available therapeutic mAbs. REGN10933 (i.e. one of the antibodies from the REGN-COV2 antibody cocktail) showed reduced activity against SARS-CoV-2 beta and gamma, although the cocktail itself (REGN10933 + 10,987) showed little change in activity against beta and gamma. However, both REGN mAbs do not exhibit any activity against SARS-CoV-2 omicron. Activity of regdanvimab was reduced against beta, gamma, and delta and completely absent against omicron. Tixagevimab did not show activity against omicron either, while cilgavimab retained some activity (albeit 15-fold lower), resulting in a 42-fold reduction of the activity of the tixagevimab/cilgavimabb cocktail against omicron. On the other hand, sotrovimab and bebtelovimab are still retaining activity against beta, delta, and omicron (Cameroni et al., 2021; Corti et al., 2021; Ryu et al., 2021; Ryu and Kang, et al., 2021; Ryu and Song, et al., 2021; Hoffmann et al., 2021; Planas et al., 2021; Westendorf et al., 2022; Ju et al., 2022; Li et al., 2022; Touret et al., 2022). This demonstrates that several of the commercially available therapeutic mAbs lose their activity against multiple SARS-CoV-2 variants of concern (VoC). In fact, mAb treatments bamlanivimab/etesevimab (LY-CoV555/LY-CoV016) from AbCellera/Eli Lilly and REGN-COV2 from Regeneron recently lost their market authorization by FDA because of no activity against SARS-CoV-2 omicron, currently the most dominant variant. Therefore, there is a need for additional potent mAbs that recognize a broad range of different SARS-CoV-2 variants.

To further broaden application and accessibility, innovations remain highly sought after in the antibody space. Gene-based delivery is one such emerging approach. Administration of the mAb sequence, using e.g. plasmid DNA (pDNA) as vector, thereby enables *in vivo* production of the mAb of interest for a prolonged period of time (Hollevoet and Declerck, 2017). Compared to conventional mAb therapy, this antibody gene transfer approach can bypass the costly and complex *in vitro* protein manufacturing, facilitate combinations, and allow for a reduced administration frequency. We previously demonstrated in mice and sheep that this technology can result in *in vivo* mAb expression for several months after intramuscular pDNA delivery, in which transfection efficiency is improved by use of electroporation (Hollevoet et al., 2018, 2019; Vermeire et al., 2021). A Phase I trial of a DNA-encoded mAb against Zika virus was initiated in 2019 (NTC: NCT03831503, sponsor: INOVIO Pharmaceuticals), further illustrating the advances in clinical translation. In the context of COVID-19, various funding organizations, including the US Defense Advanced Research Projects Agency (DARPA), have dedicated considerable funding to the development of gene-based delivery of SARS-CoV-2-neutralizing mAbs. Indeed, progress in mAb discovery and innovative delivery technologies can revolutionize emerging infectious diseases responses.

In this work, we sought to identify human mAbs reactive to the current SARS-CoV-2 VoC both *in vitro* and *in vivo*, and explore antibody gene transfer*.* We were able to generate highly potent and broadly neutralizing mAbs that are capable of treating SARS-CoV-2 Wuhan, beta, and delta infection in Syrian golden hamsters. We demonstrated efficacy both as recombinant protein and encoded in pDNA, highlighting innovation in human mAb discovery and gene-based delivery.

## Results

### Identification and characterization of anti-SARS-CoV-2 antibodies from convalescent COVID-19 patients

To identify fully human SARS-CoV-2-neutralizing antibodies, we first analyzed RBD-binding titers and neutralizing antibody titers in serum, as well as the percentage RBD-positive B cells in PBMCs from 25 convalescent COVID-19 patients (see Table S1). Patient K-COV19-901, having both a high titer of neutralizing antibodies and a high number of RBD-specific B cells, was selected for the isolation of individual RBD-specific B cells via fluorescence-activated cell sorting (FACS), using biotinylated RBD (Wuhan isolate) combined with streptavidin-PE as bait (Figure S1). Next, these single B cells were used as starting material for our single B cell cloning strategy, where we amplified the coding sequence of the IgG antibody heavy and light chain variable domains and combined them with the human IgG1 constant domain in a vector for recombinant mAb production. A panel of 20 unique mAb sequences (labeled K-COV-901-X, further mentioned as “X”) was selected for *in vitro* production and subsequent characterization. All 20 antibodies retained RBD and trimeric spike antigen (Wuhan) binding *in vitro* when analyzed via ELISA or surface plasmon resonance (SPR) assays. In addition, antibodies showed picomolar affinities to the RBD antigen and trimeric spike antigen with equilibrium constants (K_D_ values) ranging from 31 to 443 pM and 32 to 243 pM, respectively (Table 1).

**Table 1:** Overview of antigen binding, affinity, and neutralizing capacity of 20 selected antibodies

Antibody	ELISA antigen binding		Affinity K_D_ (pM)		Neutralization IC50 (nM)
	RBD (Wuhan)	Trimeric spike (Wuhan)	RBD (Wuhan)	Trimeric spike (Wuhan)	Trimeric spike (D614G)
***1A10***	***+***	***+***	***199 ± 58***	***89 ± 45***	***0.74***
1B11	+	+	443 ± 4	89 ± 18	/
***1C1***	***+***	***+***	***41 ± 12***	***44 ± 12***	***0.58***
***1C11***	***+***	***+***	***87 ± 24***	***84 ± 29***	***0.16***
1D5	+	+	111 ± 13	127 ± 78	7.37
1B5	+	+	45 ± 2	56 ± 16	1.86
2A2	+	+	220 ± 17	176 ± 88	26.22
2A8	+	+	78 ± 8	74 ± 5	8.35
***2B11***	***+***	***+***	***48 ± 21***	***32 ± 8***	***0.62***
***2B2***	***+***	***+***	***31 ± 7***	***63 ± 14***	***0.17***
2C8	+	+	64 ± 8	91 ± 15	/
2D10	+	+	52 ± 8	65 ± 36	23.38
***2D6***	***+***	***+***	***31 ± 6***	***69 ± 18***	***0.57***
3A11	+	+	58 ± 8	91 ± 18	17.37
3B3	+	+	252 ± 21	99 ± 18	/
***3B8***	***+***	***+***	***234 ± 104***	***86 ± 12***	***0.016***
3C11	+	+	48 ± 10	71 ± 11	13.71
***3E6***	***+***	***+***	***373 ± 295***	***243 ± 163***	***0.32***
3E9	+	+	248 ± 13	143 ± 20	0.97
3F9	+	+	98 ± 16	141 ± 168	/

Antigen binding was evaluated for RBD and trimeric spike protein (Wuhan) via ELISA (in duplicate) and SPR (minimum in triplicate). Affinity (K_D_; in pM; average ±SD of at least three replicates) was determined via SPR. Neutralizing capacity is given in 50% inhibitory concentration (IC_50_; in nM) and was measured in triplicate via a SARS-CoV-2 prototype (D614G) VSV pseudovirus assay. Bold and italic = 8 best neutralizing antibodies; + = potent antigen binding (OD ≥ 2) at mAb concentration of 1 μg/mL or lower; / = no neutralizing activity when tested at a concentration of 5 μg/mL or 33.34 nM.

Next, we evaluated the functional activity of these antibodies *in vitro*. To this end, we used a pseudovirus assay with vesicular stomatitis virus (VSV) expressing the SARS-CoV-2 prototype spike protein (D614G) on its surface and evaluated pseudovirus infection of Vero E6 cells in the presence and absence of each of the antibodies. Out of 20 mAbs, 16 could neutralize SARS-CoV-2 pseudovirus infection, with eight very potent mAbs having a half-maximal 50% inhibitory concentration (IC_50_) lower than 0.75 nM and mAb 3B8 having an IC_50_ of only 0.016 nM (Table 1). Given their potent neutralizing capacity, these eight mAbs were selected for further characterization.

### Evaluation of *in vitro* efficacy against SARS-CoV-2 variants

With the continuous emergence of new SARS-CoV-2 variants, it was crucial to evaluate antigen binding and neutralizing capacity of the selected antibodies against these variants as well. RBD antigens bearing the single mutations E484K, E484Q, N501Y and L452R, present—alone or combined—in multiple SARS-CoV-2 variants (i.e. alpha, beta, gamma, delta, omicron, and others), as well as RBD antigens bearing the double mutation E484Q, L452R, or triple mutation K417N, E484K and N501Y, as present in SARS-CoV-2 variants kappa and beta, respectively (CDC, 2021), were used to assess binding and affinity of the eight most potent antibodies via both ELISA and SPR. In addition, RBD mutations N439K, described as an antibody evasion mutant (J. Chen et al., 2021a; Sanyaolu et al., 2021; Thomson et al., 2021), and RBD Y453F, originating from Danish mink farms and a potential neutralization resistance mutation (Baum and Fulton, 2020; Lassaunière et al., 2021), were evaluated (an overview of the analyzed RBD single mutations and their presence in SARS-CoV-2 variants is given in Table S2). Interestingly, seven out of eight antibodies retained their binding capacity against all analyzed RBD single mutant antigens. One mAb, 2B11, showed reduced binding to RBD L452R and did not bind RBD E484K. This mAb did neither bind the antigens with multiple mutations that included L452R or E484K. mAb 1C11, although binding with all single mutant antigens, lost its activity against RBD L452R, E484Q double mutant antigen (Table 2 top part). All antibodies that bound the single mutant RBD antigens, still showed high affinities to these antigens, as evident from the equilibrium constants (K_D_) ranging from 9 to 647 pM. Only antibodies 1C11 and 2B11 showed an increased K_D_ value for RBD L452R (2940 and 3146 pM, respectively) (Table 2 bottom part).

**Table 2:** Overview of binding and affinity of top eight mAbs with RBD mutant antigens

	mAb	1A10	1C1	1C11	2B11	2B2	2D6	3B8	3E6
**ELISA antigen binding**	**Wuhan**	+	+	+	+	+	+	+	+
	**N439K**	+	+	+	+	+	+	+	+
	**L452R**	+	+	+	±	+	+	+	+
	**Y453F**	+	+	+	+	+	+	+	+
	**E484K**	+	+	+	/	+	+	+	+
	**E484Q**	+	+	+	+	+	+	+	+
	**N501Y**	+	+	+	+	+	+	+	+
	**L452R;E484Q**	+	+	/	/	+	+	+	+
	**K417N, E484K, N501Y**	+	+	+	/	+	+	+	+
		**1A10**	**1C1**	**1C11**	**2B11**	**2B2**	**2D6**	**3B8**	**3E6**
**Affinity K**_**D**_**(pM)**	**Wuhan**	199 ± 58	41 ± 12	87 ± 24	48 ± 21	31 ± 7	31 ± 6	234 ± 104	373 ± 295
	**N439K**	379 ± 120	28 ± 14	107 ± 51	42 ± 13	16 ± 5	43 ± 51	144 ± 57	299 ± 295
	**L452R**	118 ± 19	9 ± 1	2940 ± 2020	3146 ± 737	145 ± 10	18 ± 1	56 ± 3	178 ± 75
	**Y453F**	170 ± 61	36 ± 18	142 ± 143	29 ± 12	23 ± 3	20 ± 6	136 ± 49	281 ± 173
	**E484K**	106 ± 33	23 ± 7	382 ± 116	–	450 ± 37	22 ± 7	119 ± 11	122 ± 86
	**E484Q**	101 ± 22	14 ± 3	167 ± 7	647 ± 347	174 ± 18	18 ± 2	105 ± 6	87 ± 49
	**N501Y**	151 ± 64	33 ± 19	118 ± 80	32 ± 17	19 ± 5	18 ± 5	159 ± 60	162 ± 159

Antigen binding was evaluated for RBD mutant antigens via ELISA and SPR. Affinity (K_D_; in pM) was determined via SPR, mean ± SD of minimum three replicates is given. + = maximal signal obtained at mAb concentration of 1 μg/mL or lower; ± = ± 50% of the maximal signal was obtained at 1 μg/mL; / = no signal was obtained at mAb concentration of 1 μg/mL; - = no binding at RBD antigen concentration of 80 nM. See also Table S2.

**Figure 1:**
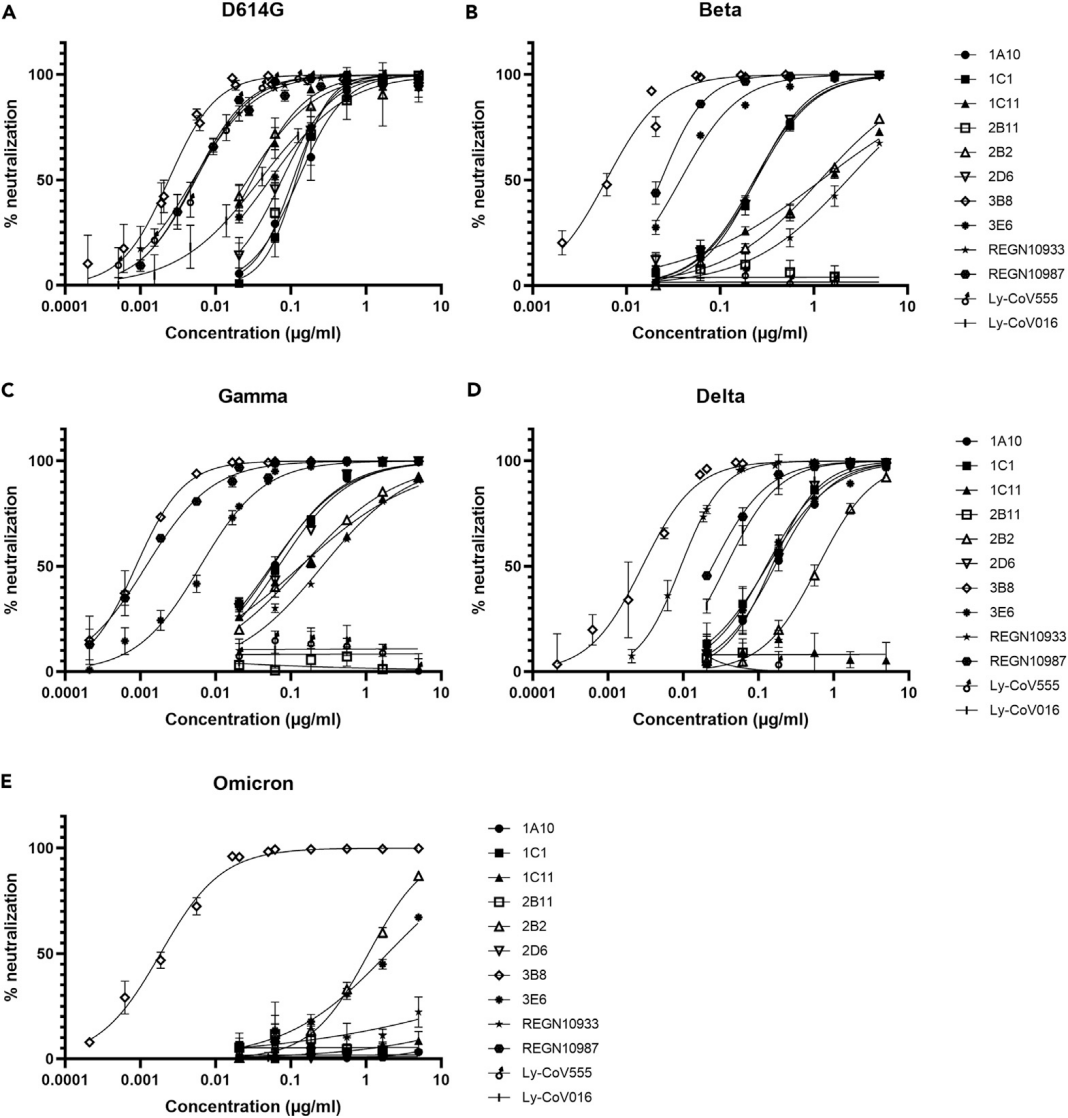
*In vitro* neutralization of infection with different SARS-CoV-2 variants (A–E). Concentration-dependent neutralizing activity (% neutralization) was measured via VSV pseudotyped assays for SARS-CoV-2 prototype (D614G) (A), beta (B), gamma (C), delta (D), or omicron (E) for the eight most potent mAbs as well as four commercial mAbs from Regeneron and Eli Lilly. Symbols show mean and SD of three replicates, graphs are representative for two independent experiments.

In addition to antigen binding, functional activity against SARS-CoV-2 variants was evaluated in a VSV pseudotyped assay for SARS-CoV-2 beta, gamma, delta, and omicron virus strains for our top eight mAbs, as well as for four commercially available clinical mAbs from Regeneron and Eli Lilly. SARS-CoV-2 prototype (D614G) was included as reference. Infection with SARS-CoV-2 beta and gamma pseudoparticles was best neutralized by antibodies 3B8 and 3E6 (IC_50_ ≤ 0.14 nM and IC_50_ ≤ 0.04 nM, for beta and gamma, respectively). SARS-CoV-2 delta was most potently neutralized by 3B8 (IC_50_ = 0.02 nM), with next in line antibodies 1A10, 1C1, 2D6, and 3E6 (IC_50_ values between 0.9 and 1.3 nM). On the other hand, antibody 2B11 was not active against any of the variants analyzed, and antibody 1C11 lost its neutralizing activity against SARS-CoV-2 delta (IC_50_ > 33 nM). Only three mAbs (2B2, 3B8, and 3E6) were able to neutralize SARS-CoV-2 omicron, with 3B8 clearly being the most potent one (IC_50_ = 0.018 nM). Overall, mAb 3B8 was the most potent neutralizing mAb toward all variants, with IC_50_ values ranging from 0.03 to 0.006 nM (Figure 1 and Table 3).

**Table 3:** Overview of *in vitro* neutralizing activity (IC_50_ and IC_90_) toward SARS-CoV-2 prototype (D614G), beta, gamma, delta, and omicron for the top eight mAbs and four commercial mAbs

Antibody	Neutralization (nM)									
	D614G		Beta		Gamma		Delta		Omicron	
	IC50	IC90	IC50	IC90	IC50	IC90	IC50	IC90	IC50	IC90
1A10	0.74	4.06	1.49	6.91	0.43	4.67	1.25	7.36	/	/
1C1	0.58	3.75	1.52	6.98	0.37	4.15	1.02	5.68	/	/
1C11	0.16	0.82	7.41	259.53	0.74	44.83	/	/	/	/
2B11	0.62	2.06	/	/	/	/	/	/	/	/
2B2	0.17	1.23	7.26	100.60	0.77	19.63	3.68	23.91	5.58	39.87
2D6	0.57	4.61	1.51	7.32	0.39	3.97	0.93	4.80	/	/
3B8	0.016	0.068	0.030	0.10	0.006	0.028	0.020	0.12	0.015	0.10
3E6	0.32	3.28	0.14	1.43	0.04	0.38	1.07	6.89	7.43	261.21

REGN10933	0.034	0.30	10.85	143.08	1.42	25.00	0.055	0.19	/	/
REGN10987	0.026	0.28	0.074	0.72	0.007	0.080	0.25	3.56	/	/
Ly-CoV555	0.036	0.18	/	/	/	/	/	/	/	/
Ly-CoV016	0.25	3.87	/	/	/	/	0.10	0.57	/	/

Neutralizing activity (IC_50_ and IC_90_; in nM) was determined via VSV pseudotyped assay. / = no neutralizing activity when tested at a concentration of 33.34 nM. IC_90_ values >33.34 nM are obtained via extrapolation of the neutralization curves.

### Classification of mAb epitopes

It has been reported that four different classes can be distinguished for SARS-CoV-2-neutralizing mAbs based on structural analysis of their epitopes. Class 1 and class 2 mAbs have epitopes overlapping with the ACE2-binding site, class 3 mAbs bind outside the ACE2-binding site, and class 4 mAbs bind a cryptic epitope outside the receptor-binding motive (RBM) of RBD (Barnes et al., 2020; Greaney et al., 2021).

**Figure 2:**
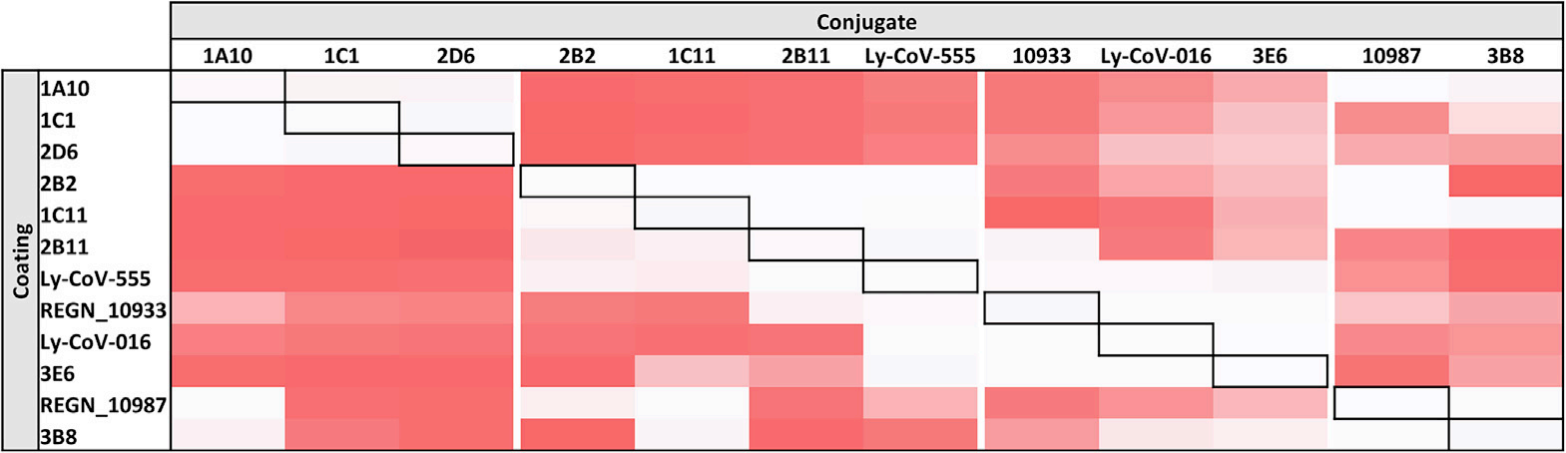
Epitope specificity of top eight mAbs and Ly-CoV016, Ly-CoV555, REGN10933, and REGN10987 Visual representation of the cross-competition ELISA. All antibodies were pairwise tested against one another as coating or detection mAb. Signal strength is visualized by color intensity, with a white color indicating no binding of the detection mAb could occur, and dark red indicating strong binding of the detection mAb. Data are representative for two independent experiments.

To classify our top eight mAbs in different categories based on their epitope specificity, we used a sandwich-type epitope binning assay where we included our in-house developed mAbs as well as Ly-CoV016, Ly-CoV555, REGN10987, and REGN10933, which are described to belong to classes 1, 2, 3 or 1&2, respectively (Barnes et al., 2020; Greaney et al., 2021). Four groups could be distinguished based on these data (Figure 2). A first group consisted of mAbs 1A10, 1C1 and 2D6, mAbs 2B2, 2B11, and 1C11 clustered together with Ly-CoV555 (corresponding to class 2), 3E6 clustered with Ly-CoV016 and REGN10933 (corresponding to class 1 and/or2), and 3B8 showed a similar competition profile as REGN10987 (corresponding to class 3). It is important to note that although the included commercial mAbs can be clustered together with some of our in-house developed mAbs, epitope binning profiles are never identical, suggesting that the epitopes are (partially) overlapping but not identical.

### Potent *in vivo* efficacy on different SARS-CoV-2 variants in the Syrian golden hamster model

**Figure 3:**
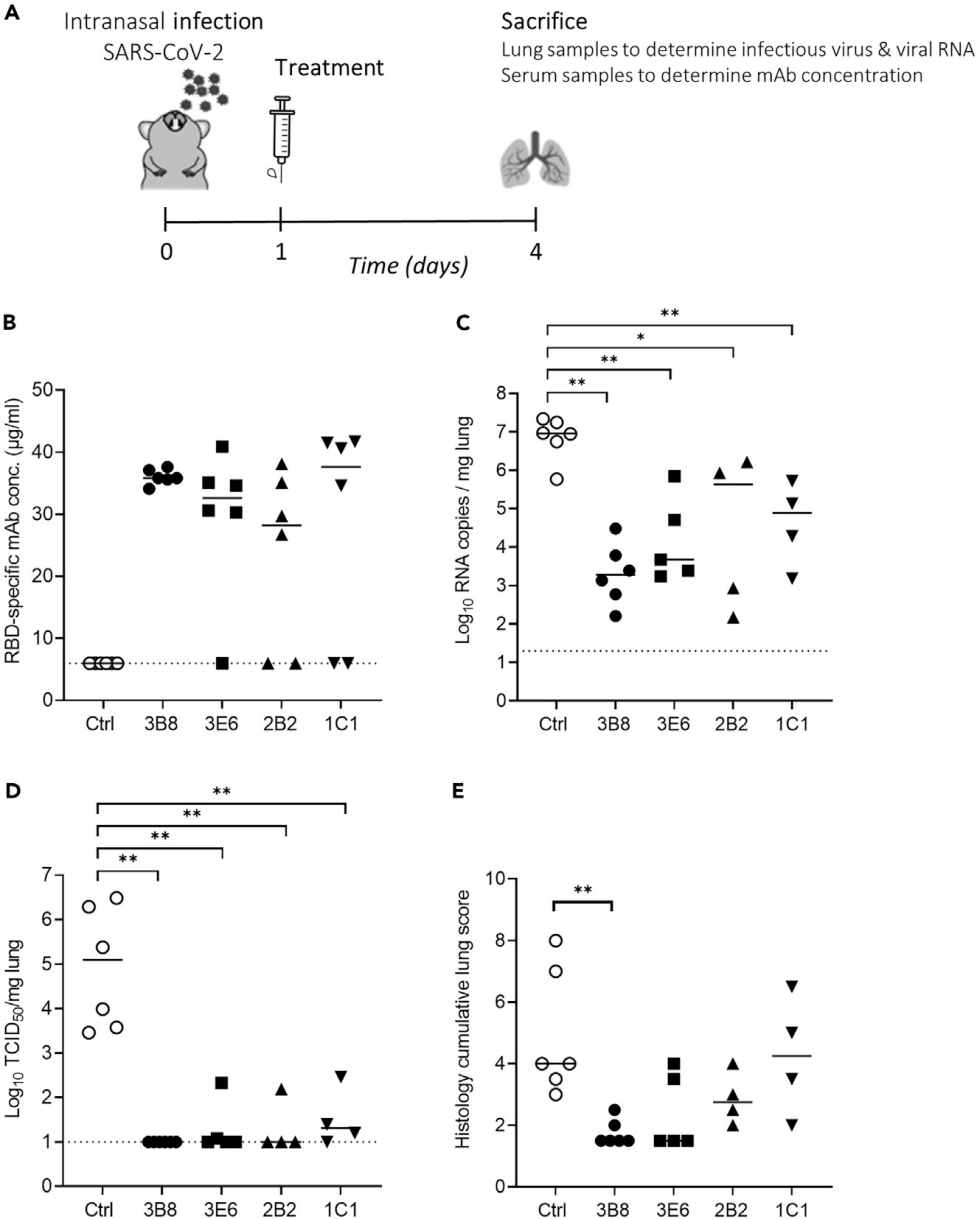
Treatment of SARS-CoV-2 Wuhan infection by antibodies 3B8, 3E6, 2B2, and 1C1 (5 mg/kg) in hamsters (A) Study design. Hamsters were intranasally infected with SARS-CoV-2 on day 0 and received intraperitoneal mAb treatment (5 mg/kg) 24 h post infection. Animals were sacrificed at day 4 for analysis of lung and blood samples. (B) Concentration (μg/mL) of the administered antibodies in serum at day 4 post infection (n = 6 per group). Animals without detectable mAb serum levels were excluded from the graphs in panel C-D-E. (C) Viral RNA levels, expressed as log_10_ RNA copies per mg of lung tissue, at day 4 post infection. (D) Infectious viral loads, expressed as log_10_ TCID_50_ per mg lung tissue, at day 4 post infection. (E) Cumulative severity score from H&E stained slides of lungs from hamsters, at day 4 post infection. Individual data with median values are shown. Dotted line represents the detection limit. Results are from one experiment. ∗ = p < 0.05; ∗∗ = p < 0.01 as determined via Mann-Whitney U test. See also Table S3.

Antibodies 3B8, 3E6, 2B2, and 1C1 (Table S3) were selected for *in vivo* evaluation based on their *in vitro* neutralizing activity combined with their variability in epitope binding. In a first study, 30 hamsters were divided into five groups (6 animals/group) and infected intranasally with 50 μL of SARS-CoV-2 Wuhan virus suspension (containing approximately 2 x 10^6^ tissue culture infective dose (TCID_50_)). After 24 h, antibodies 3B8, 3E6, 2B2, 1C1, or a human IgG1 isotype control were injected intraperitoneally at a dose of 5 mg/kg. All animals were sacrificed at 4 days post infection for analysis of viral RNA and viral infectious titer in the lungs, as well as mAb concentration in serum (Figure 3A). The presence of therapeutic RBD-specific human antibodies circulating in the blood of the treated hamsters was verified through ELISA. This revealed that five animals (1 out of six for clone 3E6 and two out of six for clones 2B2 and 1C1) were not successfully injected (Figure 3B). We therefore excluded all animals without detectable serum levels from further analysis. In all groups, mAb treatment reduced viral RNA load in lung tissue as compared to the isotype control group with statistical significance (Figure 3C). No infectious virus in the lung tissue could be detected in any of the animals treated with mAb 3B8. In animals treated with antibodies 3E6 and 2B2, a low infectious titer was detected in one animal, while three out of four animals treated with mAb 1C1 had a low detectable titer. In all groups, the difference was statistically significant compared to the isotype control group, thus showing potent *in vivo* efficacy for the treatment of SARS-CoV-2 (Wuhan) infection (Figure 3D). In addition, lung sections of the animals were blindly evaluated for lung damage and given a cumulative score between 1 and 10 representing very limited to extensive lung damage (Figure 3E). Treatment with 1C1 did not reduce lung damage when compared to isotype control. 3E6 and 2B2 show a non-significant decrease in lung damage, while 3B8 significantly reduced the cumulative lung score.

**Figure 4:**
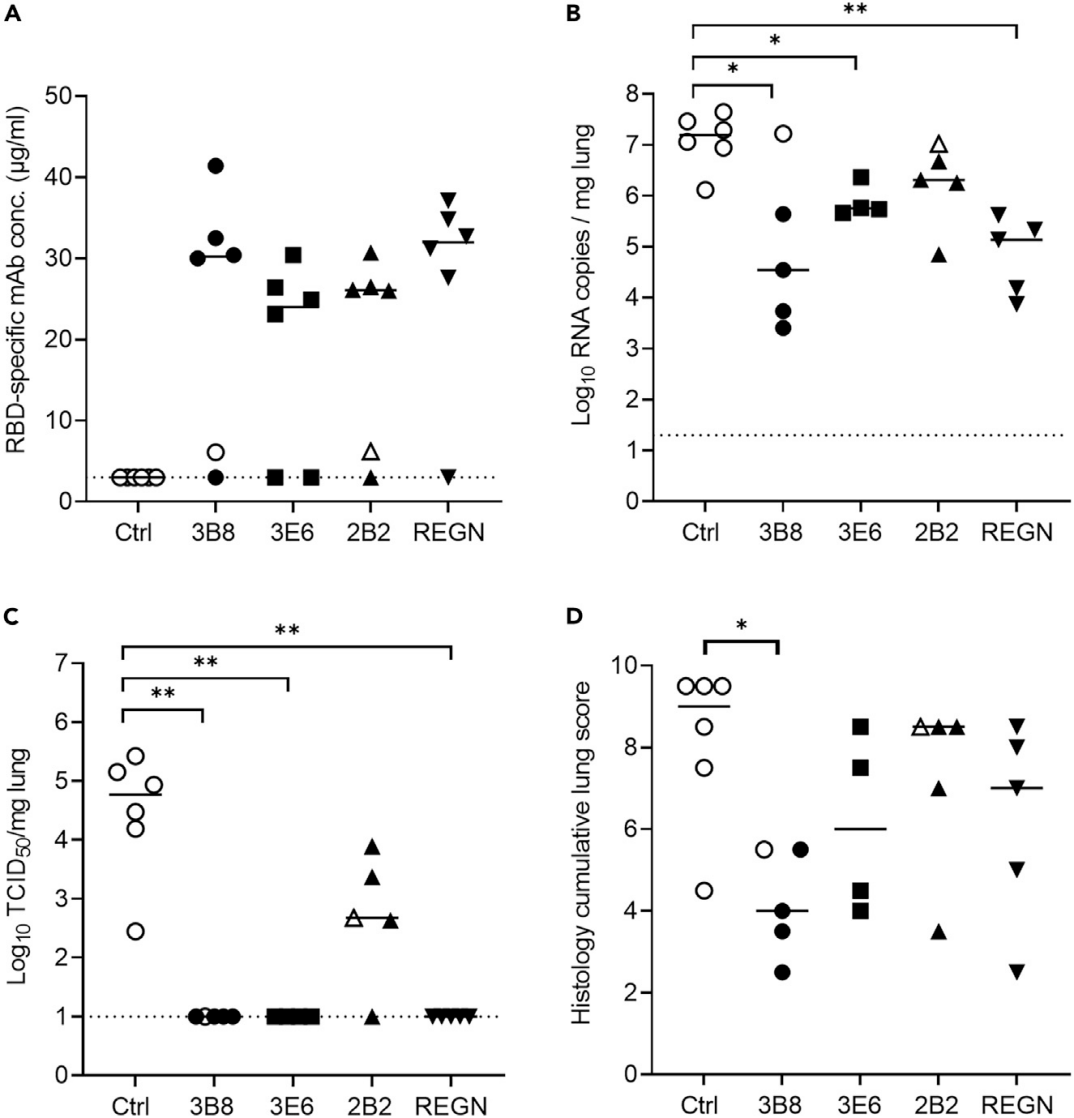
Treatment of SARS-CoV-2 beta infection by antibodies 3B8, 3E6, 2B2, and REGN-COV-2 (5 mg/kg) in hamsters (A) Concentration (μg/mL) of the administered antibodies at day 4 post infection in serum from hamsters (n = 6 per group) infected with SARS-CoV-2 beta. (B) Viral RNA levels, expressed as log_10_ RNA copies per mg of lung tissue, at day 4 post infection. (C) Infectious viral loads, expressed as log_10_ TCID_50_ per mg lung tissue, at day 4 post infection. (D) Cumulative severity score from H&E stained slides of lungs from hamsters, at day 4 post infection. Animals without detectable antibody serum levels were excluded from the graphs in panel B-C-D. Individual data and median values are shown. Dotted line represents the detection limit. Results are from one experiment. ∗ = p < 0.05; ∗∗ = p < 0.01 as determined via Mann-Whitney U test. See also Table S3.

**Figure 5:**
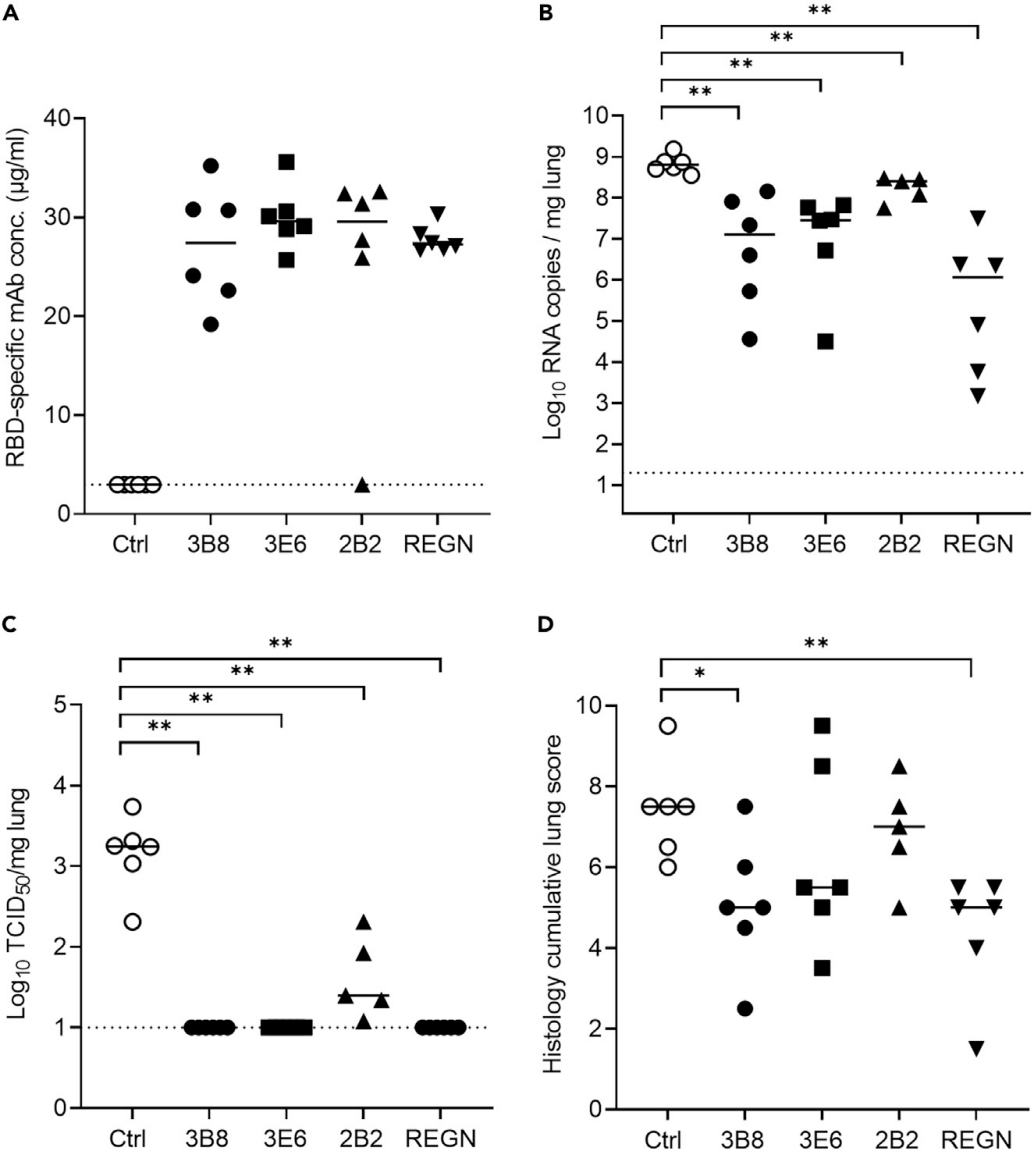
Treatment of SARS-CoV-2 delta infection by antibodies 3B8, 3E6, 2B2, and REGN-COV-2 (5 mg/kg) in hamsters (A) Concentration (μg/mL) of the administered antibodies at day 4 post infection in serum from hamsters (n = 6 per group) infected with SARS-CoV-2 delta. (B) Viral RNA levels, expressed as log_10_ RNA copies per mg of lung tissue, at day 4 post infection. (C) Infectious viral loads, expressed as log_10_ TCID_50_ per mg lung tissue, at day 4 post infection. (D) Cumulative severity score from H&E stained slides of lungs from hamsters, at day 4 post infection, see also Figure S2. Animals without detectable antibody serum levels were excluded from the graphs in panel B-C-D. Individual data and median values are shown. Dotted line represents the detection limit. Results are from one experiment. ∗ = p < 0.05; ∗∗ = p < 0.01 as determined via Mann-Whitney U test. See also Table S3.

As it was shown previously that SARS-CoV-2 VoC beta and delta are more resistant to neutralization by vaccine-induced antibodies or currently available mAb treatments (Wang et al., 2021a; Planas et al., 2021; Zhou et al., 2021), we decided to evaluate *in vivo* treatment efficacy of our antibodies against both variants as well. A study design similar to the first study was used, with 30 hamsters divided in five groups and intranasally infected with 50 μL of SARS-CoV-2 beta (Abdelnabi et al., 2021) or SARS-CoV-2 delta suspension (both containing approximately 1 x 10^4^ TCID_50_). Animals were injected intraperitoneally with IgG1 isotype control or antibodies 3B8, 3E6, 2B2, and REGN-COV-2 at 5 mg/kg 24 h post infection and were sacrificed 4 days post infection, with REGN-COV-2 antibody cocktail included for benchmarking purposes (Food and Drug Administration, 2021). In the study using SARS-CoV-2 beta, one animal from groups 3B8, 2B2, 1C1, and REGN-CoV-2 as well as two animals from group 3E6 were excluded from analysis because of the absence of detectable antibody serum levels (Figure 4A). In addition, it should be noted that two animals, one from group 3B8 and one from group 2B2, showed very low antibody serum titers, but were not excluded from analysis (indicated by open symbols in the respective groups). Viral RNA load in lung tissue was significantly reduced by treatment with mAb 3B8, 3E6, and REGN-CoV-2 compared to isotype control, while the reduction observed for 2B2 was not statistically significant (Figure 4B). No infectious viral titer in lung tissue (TCID_50_) could be detected upon treatment with 3B8, 3E6, or REGN-CoV-2, while a viral titer was still detectable in four out of five animals treated with 2B2 (Figure 4C). 3B8 significantly (p < 0.05) reduced the cumulative lung score, while all other treatments only show a trend toward reduced cumulative lung score (Figure 4D). When analyzing the efficacy of our antibodies against SARS-CoV-2 delta infection, a similar picture was observed. Here, only one animal from group 2B2 was excluded because of a lack of detectable serum concentrations (Figure 5A). All treatments resulted in a significant reduction of both viral load and infectious viral titers in lung tissue (Figures 5B and 5C), with no detectable viral titers in animals treated with antibodies 3B8, 3E6, and REGN-CoV-2. When looking at lung histopathology, a significant decrease in cumulative lung score was observed for both 3B8 and REGN-CoV-2. 3E6 showed a trend toward decreased cumulative lung score, while 2B2 did not affect lung score (Figure 5D). In conclusion, treatment with antibodies 3B8 and 3E6 at 24 h post infection resulted in a statistically significant reduction of virus in lung tissue of animals infected with SARS-CoV-2 beta or delta, with 3B8 also significantly reducing cumulative lung score. Although 2B2 significantly reduced infectious viral titers in lung tissue of animals infected with SARS-CoV-2 delta, this mAb seems to be less potent as treatment for SARS-CoV-2 beta and delta infection compared to antibodies 3B8 and 3E6, an observation corresponding to the *in vitro* neutralization data for these variants.

### Dose-dependent therapeutic efficacy of 3B8

**Figure 6:**
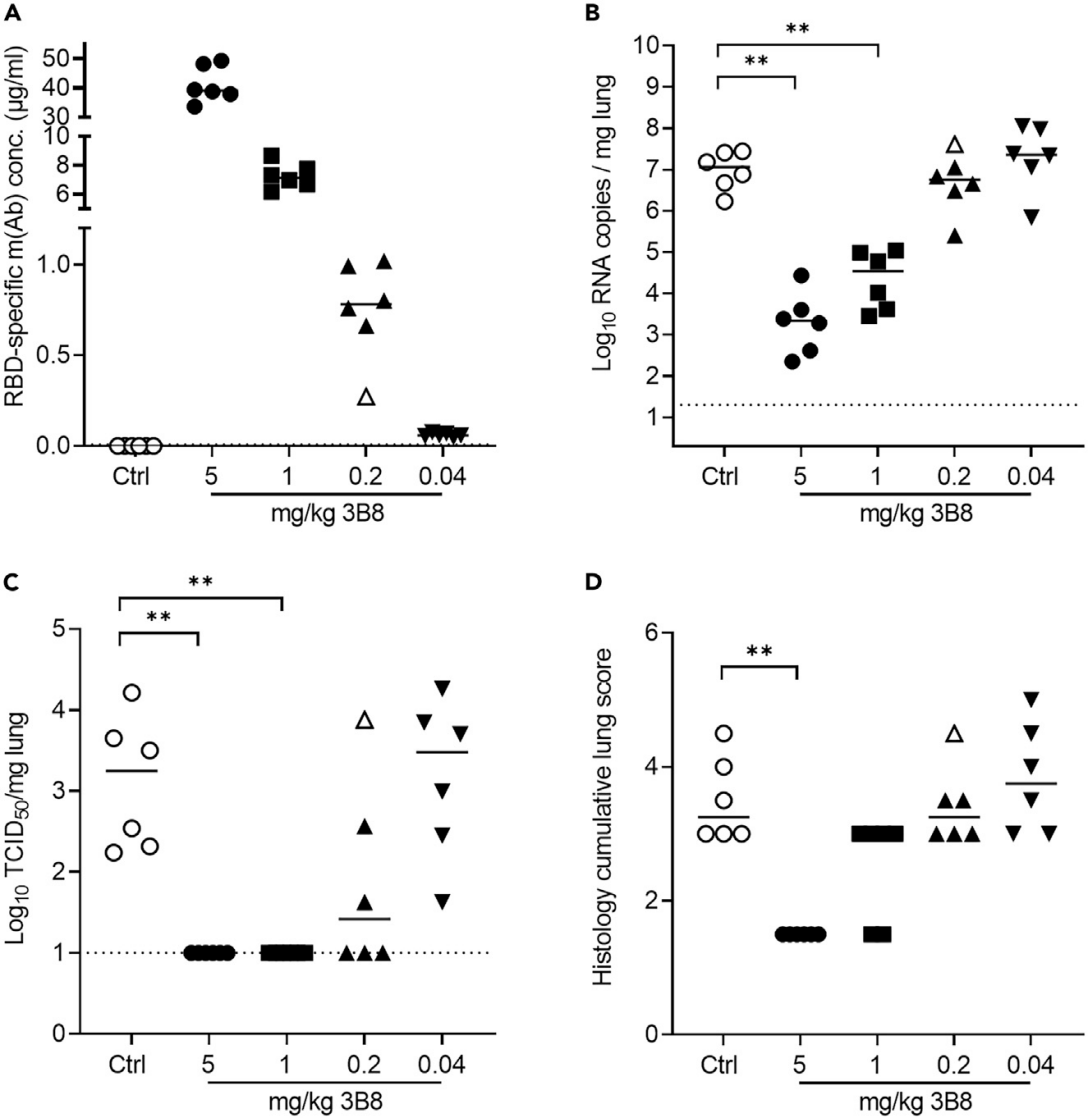
Dose-response experiment for treatment of SARS-CoV-2 delta infection with mAb 3B8 (5, 1, 0.2, or 0.04 mg/kg) in hamsters (A) Concentration (μg/mL) of the administered antibodies in serum at day 4 post infection (n = 6 per group). (B) Viral RNA levels, expressed as log_10_ RNA copies per mg of lung tissue, at day 4 post infection. (C) Infectious viral loads, expressed as log_10_ TCID_50_ per mg lung tissue, at day 4 post infection. (D) Cumulative severity score from H&E stained slides of lungs from hamsters, at day 4 post infection, see also Figure S3. Individual data and median values are shown. Dotted line represents the detection limit. Results are from one experiment. ∗∗ = p < 0.01 as determined via Mann-Whitney U test.

Antibodies 3B8 and 3E6 both completely abrogated infectious viral titers after infection with SARS-CoV-2 Wuhan, beta, and delta when administered 24 h post infection at 5 mg/kg. As 3B8 was superior to 3E6 based on the lung histopathology and *in vitro* neutralization experiments, this mAb was used in an *in vivo* dose-response experiment. Hamsters were intranasally infected with SARS-CoV-2 delta (1 x 10^4^ TCID_50_). At 24 h post infection, isotype control (5 mg/kg) or mAb 3B8 (5 mg/kg; 1 mg/kg, 0.2 mg/kg or 0.04 mg/kg) was administered intraperitoneally. Animals were sacrificed at day 4 post infection. No animals were excluded from analysis based on a lack of detectable antibody serum levels, although one animal from group 0.2 mg/kg had serum levels 3 times lower compared to its group members (animal indicated by open symbol) (Figure 6A). Treatment with a dose of 5 mg/kg and 1 mg/kg of 3B8 significantly reduced the amount of viral RNA detected in lung tissue, and resulted in undetectable levels of infectious virus. 5, 1, and 0.2 mg/kg showed significant differences in viral RNA (p < 0.05 or <0.01 for 5 vs 1 mg/kg or 5 vs 0.2/0.04 mg/kg, respectively; p < 0.01 for 1 vs 0.2 or 0.04 mg/kg), indicating a positive dose-response effect. When dosing at 0.2 mg/kg, no statistically significant (p = 0.13) decrease in viral RNA was seen, but infectious virus was undetectable in lung tissue of three out of six animals, and was decreased (more than 4 times lower than lower limit of 95% confidence interval from isotype control group) in a fourth animal. In the animal having reduced antibody titers (open symbol), no reduction in viral titer could be observed. Also, at the lowest dose of 0.04 mg/kg, no effect could be observed on viral RNA or infectious viral titer (Figures 6B and 6C). At 5 mg/kg, treatment with 3B8 resulted in significantly reduced cumulative lung score. When dosed at 1 mg/kg, cumulative lung score was reduced in two out of six animals, with no detectable reduction at a dose of 0.2 or 0.04 mg/kg (Figure 6D).

### Intramuscular delivery of DNA-based 3B8

**Figure 7:**
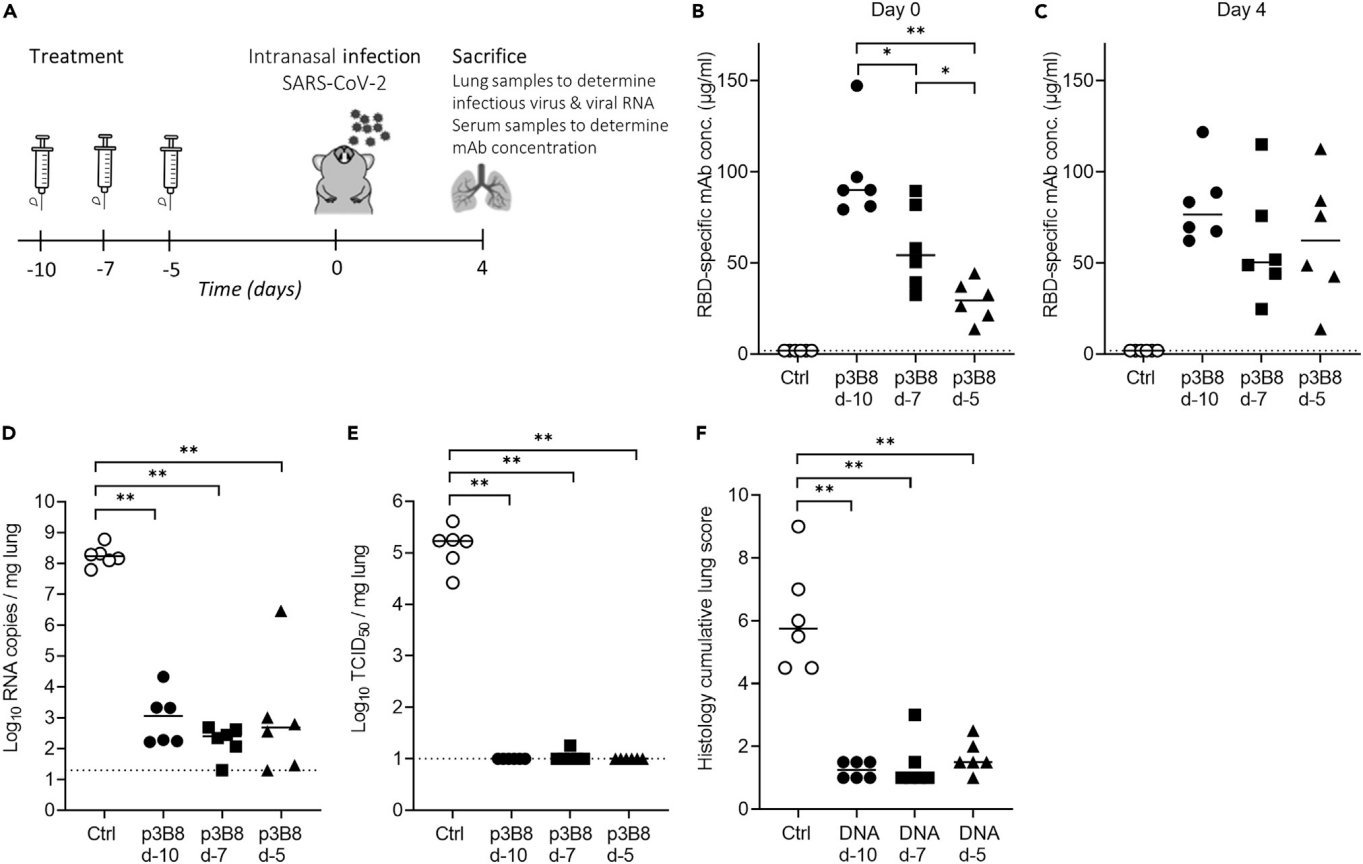
Pharmacokinetics and efficacy of intramuscular DNA-encoded 3B8 delivery for protection against SARS-CoV-2 delta infection in hamsters (A) Study design. 600 μg p3B8 was delivered via intramuscular electroporation at either 10, 7 or 5 days prior to infection (n = 6 per group). Animals of the negative control group were left untreated. Concentration (μg/mL) of 3B8 in serum at day 0 (B) and 4 (C) post infection. (D) Viral RNA levels, expressed as log_10_ RNA copies per mg of lung tissue, at day 4 post infection. (E) Infectious viral loads, expressed as log_10_ TCID_50_ per mg lung tissue, at day 4 post infection. (F) Cumulative severity score from H&E stained slides of lungs from hamsters, at day 4 post infection. Individual data and median values are shown. Dotted line represents the detection limit. Results are from one experiment. ∗ = p < 0.05; ∗∗ = p < 0.01 as determined via Mann-Whitney U test.

To assess whether 3B8 could be produced *in vivo* at sufficient titers to protect from SARS-CoV-2 delta infection, intramuscular electroporation of DNA-encoded 3B8 (p3B8) was performed in hamsters either on day 10, day 7, or day 5 prior to infection (Figure 7A). Median 3B8 serum levels at day 0, the day of intranasal infection, ranged between 30 μg/mL (p3B8 at day −5) and 90 μg/mL (p3B8 at day −10) (Figure 7B). All transfected hamsters displayed detectable mAb titers. The longer the lag time between p3B8 delivery and infection, the higher the resulting serum 3B8 concentrations at day 0. This was anticipated, as *in vivo*-expressed mAb levels typically increase and accumulate in the first two weeks after intramuscular pDNA administration (Hollevoet et al., 2018; Vermeire et al., 2021). Of note, mAb titers did not consistently or significantly increase between day 0 and day 4 post infection, and a markedly higher variability was observed at day 4 (Figure 7C). These observations are likely linked to the interaction of 3B8 with the virus (including target-mediated clearance) from day 0 on. Irrespective of the timing of pDNA administration, the *in vivo*-produced 3B8 levels were sufficient to protect the animals from viral challenge. Compared to the untreated control group, all animals showed a significantly lower amount of viral RNA in lung tissue, undetectable levels of infectious virus (except for one animal at day −7) and significantly reduced cumulative lung scores (Figures 7D–7F). No 3B8 dose-response effect was observed, since all timepoints gave a profound protection. These data show how intramuscular injection of DNA-based 3B8 provides a robust *in vivo* mAb expression and protection against SARS-CoV-2 delta infection.

## Discussion

Despite vaccination rates steadily increasing, the majority of individuals worldwide have not been fully vaccinated and the number of SARS-CoV-2-related hospitalizations and deaths continues to increase (Ritchie et al., 2021). In addition, vaccine-breakthrough infections are becoming more prevalent. These may be explained by waning vaccine protection, and the emergence of SARS-CoV-2 variants with reduced sensitivity to vaccine-elicited antibody neutralization (Calcoen et al., 2022; Andrews et al., 2021; Planas et al., 2021; Chen et al., 2021b; Tartof et al., 2021;Wang et al., 2021b; Cohn et al., 2021; Collier et al., 2021; Connor et al., 2021; Goldberg et al., 2021; Hacisuleyman et al., 2021; Hoffmann et al., 2021; Kustin et al., 2021; McCallum et al., 2021). mAb treatments can therefore play a crucial role in addressing COVID-19 morbidity and mortality and to prevent rapid dissemination of viral infection in specific settings, such as elderly care homes.

In this study, eight highly potent neutralizing mAbs (IC_50_ < 0.75 nM for neutralization of SARS-CoV-2 prototype (D614G)) were identified from a convalescent COVID-19 patient, by leveraging our B cell mining platform. Six out of eight antibodies bound all analyzed recombinant SARS-CoV-2 RBD antigens bearing single or multiple mutations. Affinities were evaluated for RBD antigens with single mutations and shown to be in the picomolar range. Corresponding to the antigen binding data, *in vitro* pseudotyped neutralization assays for SARS-CoV-2 prototype (D614G), beta, gamma, and delta strains showed that these six antibodies potently cross-neutralized these variants with IC_50_ values ranging from 0.006 to 7.41 nM. In contrast, only three out of eight mAbs were able to neutralize SARS-CoV-2 omicron, with IC_50_ values between 0.015 and 7.43 nM. Interestingly, data from the cross-competition ELISA showed that these antibodies were not all targeting the same epitope, but could be divided into four epitope groups.

The most potent mAb of each of these epitope classes was tested for therapeutic activity *in vivo* in Syrian golden hamsters. This model has been widely used to assess the efficacy of vaccines and therapeutics against SARS-CoV-2 infection as it closely mimics the clinical disease observed in humans (Baum and Ajithdoss, 2020; Abdelnabi et al., 2021; Andreano et al., 2021). Using this model, we designed three different therapeutic studies to evaluate the efficacy of our antibodies as a treatment for SARS-CoV-2 Wuhan, beta, and delta infection. We showed that antibodies 3B8 and 3E6 reduced median viral RNA titers with a factor 50–5000 or 10–330, respectively, when used as a treatment (5 mg/kg) for all tested SARS-CoV-2 strains. In addition, treatment with these antibodies resulted in undetectable levels of infectious virus in the lungs of almost all animals. Our *in vitro* and *in vivo* data suggest that 3B8 and 3E6 target an epitope conserved between the different variants, abolishing the need for combination therapy for the current SARS-CoV-2 variants. In addition, since we identified potent neutralizing antibodies from different epitope groups, combination therapy remains an option in the future if additional variants of concern appear. Indeed, thanks to the advancements in mAb identification and characterization at display in this study, we can rapidly identify multiple mAbs of varying specificity and potency, which in combination could result in enhanced breadth and potency.

Importantly, the current data demonstrate that 3B8 is a potent therapeutic mAb, which results in undetectable infectious viral titers and reduced cumulative lung scores in two out of six animals at a dose of 1 mg/kg. Of note, the relatively low cumulative lung scores in the isotype control group in this experiment (median score of 3.25, compared to 7.5 and 5.75 for the other two experiments performed with SARS-CoV-2 delta) might mask further differences between treatment and control groups. Interestingly, even when administered at 0.2 mg/kg, viral replication was reduced, with median infectious viral titers of the treatment group being 67 times lower compared to the isotype control group. This further highlights the potency of mAb 3B8 in a therapeutic setting (Fagre et al., 2020; Kreye et al., 2020; Walls et al., 2020; Andreano et al., 2021; Piepenbrink et al., 2021; Winkler et al., 2021). High potencies are crucial to decrease cost of goods, enable sustainable manufacturability, and increase the number of doses produced annually, and may therefore also facilitate the availability of therapeutic mAbs to low- and middle-income countries.

When comparing the mAbs identified in this study with the commercially available ones, we noticed that K_D_ values observed for our antibodies were consistently between 3- and 200-fold lower than values reported for marketed antibodies of Vir Biotechnology, Regeneron Pharmaceuticals or Eli Lilly (Hansen et al., 2020; Jones et al., 2020; Tortorici et al., 2020). However, when analyzing K_D_ values of REGN-COV2 antibodies 10933 and 10987 in our own experiments for benchmarking purposes, K_D_ values were similar to values observed for our mAbs (data not shown). When looking at *in vitro* pseudotyped neutralization data for SARS-CoV-2 beta, gamma, and delta, we observed that IC_50_ values of 3B8 were lower than those of REGN10933 and REGN10987 and of Ly-CoV555 and Ly-CoV016, although differences were small for some variants. When looking at SARS-CoV-2 omicron neutralization, we confirmed that the mAbs from Regeneron and Eli Lilly do not exhibit neutralizing activity, while three of our mAbs (including 3B8) remained active (Table 3). In addition, it should be noted that antibodies 3B8 and 3E6 each performed equally well as monotherapy (when administered at 5 mg/kg) to treat SARS-CoV-2 infection beta and delta in hamsters when compared to the marketed REGN-COV2 antibody cocktail, which consists of two different antibodies (Food and Drug Administration, 2021). Moreover, we could observe therapeutic efficacy of mAb 3B8 *in vivo* at a dose of only 0.2 mg/kg. Only for REGN-COV2, some therapeutic efficacy was reported in hamsters at such low mAb doses, although after infection of hamsters with SARS-CoV-2 Wuhan and using different readouts (Baum et al, 2020).

In addition to demonstrating our ability to select and characterize highly potent neutralizing human mAbs from a convalescent COVID-19 patient, we evaluated the DNA-based delivery of 3B8, an innovative delivery approach that could revolutionize the application of mAb therapeutics for infectious diseases (Hollevoet and Declerck, 2017; Andrews et al., 2020). We found that intramuscular electroporation of p3B8 provided a robust *in vivo* mAb expression and protection against SARS-CoV-2 delta infection, irrespective of the timepoint of p3B8 administration. The resulting mAb serum concentrations produced *in vivo* were similar to or significantly higher than what was observed after i.p. injection of 5 mg/kg 3B8 protein. As a consequence, lower pDNA doses and/or alternative timepoints (closer to or after viral infection) are anticipated to still demonstrate efficacy. To the best of our knowledge, this is the first report to show that *in vivo* DNA-based delivery of a SARS-CoV-2-neutralizing mAb can protect from viral infection. As elaborated on earlier, gene-based delivery of mAbs is coming of age. The first clinical trials further highlight the application within infectious diseases, in a preventive and potentially also therapeutic setting. Overall, such approach can complement and accelerate the discovery, development, and delivery of (combinations of) mAbs, and allow us to keep up the pace with the current and future pandemics.

Although no clear evidence for antibody-dependent disease enhancement (ADE) in patients with COVID-19 has been reported until now, it has been suggested that its potential risk should be continuously monitored (Lee et al., 2020; Andreano et al., 2021; Corti et al., 2021; Taylor et al., 2021). In this light, it should be emphasized that the high potency of 3B8 is obtained without the contribution of Fc-mediated effector functions, as fully human IgG1 antibodies were not species-matched before administration to hamsters. This suggests that mAb 3B8 will tolerate the future introduction of Fc-effector silence mutations to avoid any potential risk of ADE, favoring its further evaluation as a future mAb treatment.

In conclusion, the high therapeutic potency of mAb 3B8 *in vivo* against SARS-CoV-2 delta, achieved both as protein and encoded in plasmid, combined with its broad cross-reactivity against all other tested SARS-CoV-2 variants, make 3B8 a very interesting candidate to help fight the COVID-19 pandemic. The combination of accelerated human mAb discovery and innovative gene-based delivery, at display in the current study, holds the potential to revolutionize emerging infectious diseases responses.

### Limitations of the study

While we included commercially available mAbs from Regeneron and Eli Lilly in the performed *in vitro* experiments to compare with our mAbs, the number of approved therapeutic mAbs continuously evolves, making it unfeasible to include all of them.

Secondly, although *in vitro* neutralization experiments and *in vivo* studies in hamsters showed that our mAbs are very potent, these models can never fully predict efficacy in humans. To determine the therapeutic potential of our mAbs in patients, clinical trials are required.

## STAR★Methods

### Key resources table

**Table:** 

REAGENT or RESOURCE	SOURCE	IDENTIFIER
**Antibodies**		

PerCP-cy5.5 anti-human CD19 antibody	Biolegend	Cat# 363016, clone SJ25C1; RRID:AB_2564207
FITC anti-human CD3 antibody	Biolegend	Cat# 300306, clone HIT3a; RRID:AB_314042
anti-SARS-CoV-2 RBD mAb	Sino Biological	Cat# 40150-D004; RRID:AB_2827983
anti-SARS-CoV-2 nucleocapsid antibody	MyBioSource	Cat# MBS2563841

**Bacterial and virus strains**		

SARS-CoV-2 Wuhan (BetaCov/Belgium/GHB-03021/2020; EPI ISL 109 407976|2020-02-03)	Prof. Piet Maes; https://doi.org/10.1038/s41467-022-28354-0	PMID: 33037151
SARS-CoV-2 Beta B.1.351 (hCoV-19/Belgium/rega-1920/2021; EPI_ISL_896474, 2021-01-11)	Prof. Piet Maes; https://doi.org/10.1038/s41467-022-28354-0	PMID: 34049240
SARS-CoV-2 Delta B.1.617.2 (hCoV-19/Belgium/rega-7214/2021; EPI_ISL_2425097; 2021-04-20)	Prof. Piet Maes; https://doi.org/10.1038/s41467-022-28354-0	PMID: 35169114

**Biological samples**		

Human COVID-19 patient PBMCs	This paper	N/A
Human COVID-19 patient serum	This paper	N/A

**Chemicals, peptides, and recombinant proteins**		

PE streptavidin	Biolegend	Cat# 405203
Spike glycoprotein (S1) RBD-His	The Native Antigen Company	Cat# REC31849-500
RBD(N439K)-His	Sino Biological	Cat# 40592-V08H14
RBD(N501Y)-His	Sino Biological	Cat# 40592-V08H82
RBD(E484K)-His	Sino Biological	Cat# 40592-V08H84
RBD(Y453F)-His	Sino Biological	Cat# 40592-V08H80
RBD(E484Q)-His	Sino Biological	Cat# 40592-V08H81
RBD(L425R)-His	Sino Biological	Cat# 40592-V08H28
RBD(L425R,E484Q)-His	Sino Biological	Cat# 40592-V08H88
RBD(K417N, E484K, N501Y)-His	Sino Biological	Cat# 40592-V08H85
Spike Glycoprotein (Full-Length)-His	The Native Antigen Company	Cat# REC31868-500
Zombie Aqua Fixable Viability Kit	Biolegend	Cat# 423101
FcR blocking reagent	Miltenyi Biotec	Cat# 130-059-901
KAPA HiFi HotStart ReadyMix	Roche	Cat# 103568-586

**Critical commercial assays**		

EasySep™ Human B Cell Enrichment Kit	Stem Cell Technologies	Cat# 17954
EZ-Link Sulfo-NHS-LC-Biotin kit	Thermo Fisher	Cat# 21335
Agencourt AMPure XP beads	Beckman Coulter	Cat# A63880
NucleoBond Xtra Maxi EF kit	Machery - Nagel	Cat# 740424.50

**Experimental models: Cell lines**		

FreeStyle™ 293-F Cells	Thermo Fisher	Cat# R79007
HEK293T cells	ATCC	ATCC Cat# CRL-3216, RRID:CVCL_0063
Vero E6 cells	ATCC	ATCC Cat# CRL-1586
BHK-21J	https://doi.org/10.1128/JVI.71.12.96089617.1997	N/A

**Experimental models: Organisms/strains**		

Golden Syrian Hamsters (*Mesocricetus auratus*)	Janvier Laboratories	Strain name RjHan:AURA

**Oligonucleotides**		

oligo-dT primer (5′–AAGCAGT GGTATCAACGCAGAGTACT_30_-3′)	IDT DNA	N/A
Forward primer (described by Picelli et al.) (5′-AAGCAGTGGT ATCAACGCAGAGT-3′)	IDT DNA	N/A
Reverse primer IgM variable region (described by Ozawa et al.) (5′-CCGACGGGGAA TTCTCACAG-3′)	IDT DNA	N/A
Reverse primer IgG variable region (described by Ozawa et al.) (5′-CGCCTGAGTTCC ACGACACC-3′)	IDT DNA	N/A
Reverse primer Kappa variable region (described by Ozawa et al.) (5′-GAGGCAGTTCCA GATTTCAA-3′)	IDT DNA	N/A
Reverse primer Lamda variable region (described by Ozawa et al.) (5′-GCTT GGAGCTCCTCAGAGG-3′)	IDT DNA	N/A

**Recombinant DNA**		

VSVΔG	https://doi.org/10.1016/j.jviromet.2010.08.006	N/A
D614G Spike variant	https://doi.org/10.1038/s41586-020-3035-9	N/A
Beta Spike variant	Invivogen	plv-spike-v3
Gamma Spike variant	Invivogen	plv-spike-v5
Delta Spike variant	Invivogen	plv-spike-v8
Omicron Spike variant	https://doi.org/10.1101/2021.11.12.468374	N/A

**Software and algorithms**		

GraphPad Prism		N/A
Biacore T200 Evaluation Software 3.1		N/A
Thermo Fisher Scientific HCS Studio (v.6.6.0) software		N/A

### Resource availability

#### Lead contact

Further information and requests for resources and reagents should be directed to and will be fulfilled by the lead contact, Dr. Nick Geukens (nick.geukens@kuleuven.be).

#### Materials availability

There are restrictions to the availability of the human monoclonal antibodies discovered and characterized in this study due to IP.

### Experimental model and subject details

#### Human subjects

COVID-19 patients with a PCR-confirmed SARS-CoV-2 infection (>18 years old; recovered from disease) were recruited from the University Hospital Leuven or AZ Groeninge Kortrijk for peripheral blood collection (sample size 25; age 25-81yearears; 18 male and 7 female; see Table S1). The study and corresponding experiments were approved by the local ethics committee (S64089) and all patients gave their written informed consent.

#### Animals

Female Syrian hamsters (*Mesocricetus auratus*) were purchased from Janvier Laboratories and kept per two in individually ventilated isolator cages (IsoCage N Bio-containment System, Tecniplast) at 21°C, 55% humidity and 12:12 day/night cycles. At the time of the experiments, animals were 6-8 weeks old and weighed 75-100g. Housing conditions and experimental procedures were approved by the ethics committee of animal experimentation of KU Leuven (license P065-2020).

#### Cell lines

##### HEK293-F cells

FreeStyle™ 293-F Cells (Human Embryonic Kidney, ThermoFisher, cat nr R79007) were cultured in Freestyle 293 expression medium (Gibco) and kept in Erlenmeyer flasks in a 37°C incubator containing a humidified atmosphere of 8% CO_2_ on an orbital shaker platform rotating at 135 rpm.

##### HEK293-T cells

HEK293-T cells (Human Embryonic Kidney, ATCC CRL-3216) were maintained in Dulbecco’s modified Eagle medium (DMEM, Gibco), supplemented with 10% fetal bovine serum (Hyclone) and 100 units/ml penicillin–streptomycin solution (Pen/Strep, Gibco). For pseudotype production medium contained 2% heat-inactivated fetal bovine serum. Cells were kept in a humidified 5% CO_2_ incubator at 37°C.

##### BHK-21J cells

BHK-21J (Baby Hamster Kidney fibroblasts) cells (Lindenbach and Rice, 1997) were provided by P. Bredenbeek and maintained in minimum essential medium (MEM, Gibco), supplemented with 10% fetal bovine serum (Hyclone), 1% non-essential amino acids (NEAA, Gibco), 100 units/ml penicillin–streptomycin solution (Pen/Strep, Gibco), 2 mM L-glutamine (Gibco), 1% sodium bicarbonate (Gibco) and 0.01 M HEPES (Gibco). For pseudotype production medium contained 2% heat-inactivated fetal bovine serum. Cells were kept in a humidified 5% CO_2_ incubator at 37°C.

##### Vero E6 cells

Vero E6 cells (African green monkey kidney, ATCC CRL-1586) were cultured in minimal essential medium (MEM, Gibco) supplemented with 10% fetal bovine serum (Integro or Hyclone), 1% non-essential amino acids (NEAA, Gibco), 100 units/ml penicillin–streptomycin solution (Pen/Strep, Gibco), 2 mM L- glutamine (Gibco) , 0.01 M HEPES (Gibco) and 1% bicarbonate (Gibco). End-point titrations on Vero E6 cells were performed with medium containing 2% fetal bovine serum instead of 10%. Cells were kept in a humidified 5% CO_2_ incubator at 37°C.

#### Viral strains

Three SARS-CoV-2 strains were used in this study. BetaCov/Belgium/GHB-03021/2020 (EPI ISL 109 407976|2020-02-03), was recovered from a nasopharyngeal swab taken from an RT-qPCR confirmed asymptomatic patient who returned from Wuhan, China in the beginning of February 2020. A close relation with the prototypic Wuhan-Hu-1 2019-nCoV (GenBank accession 112 number MN908947.3) strain was confirmed by phylogenetic analysis. Infectious virus was isolated by serial passaging on Huh7 and Vero E6 cells (Kaptein et al., 2020); passage 6 virus was used for the study described here. The Beta variant B.1.351 (hCoV-19/Belgium/rega-1920/2021; EPI_ISL_896474, 2021-01-11) was isolated from nasopharyngeal swabs taken from a traveler returning to Belgium in January 2021 who became a patient with respiratory symptoms. The Delta variant, B.1.617.2 (hCoV-19/Belgium/rega-7214/2021; EPI_ISL_2425097; 2021-04-20) was isolated from nasopharyngeal swabs in April 2021 in Belgium through active surveillance. Both strains were subjected to sequencing on a MinION platform (Oxford pore) directly from the nasopharyngeal swabs. Infectious virus was isolated by passaging on Vero E6 cells (Abdelnabi et al., 2021); passage 2 was used for the study described here. Live virus-related work was conducted in the high-containment A3 and BSL3+ facilities of the KU Leuven Rega Institute (3CAPS) under licenses AMV 30112018 SBB 219 2018 0892 and AMV 23102017 SBB 219 20170589 according to institutional guidelines.

### Method details

#### Isolation of PBMCs from convalescent COVID-19 patients

Immediately after blood sample collection of human COVID-19 patients, peripheral blood mononuclear cells (PBMCs) were isolated from EDTA-treated blood by density centrifugation (Lymphoprep, STEMCELL Technologies Inc). After washing, the collected PBMCs were resuspended in freezing medium consisting of 10% (v/v) dimethyl sulfoxide (DMSO; Merck) and 90% fetal bovine serum (FBS; GIBCO) for cryopreservation at −80°C or in liquid nitrogen for long term storage.

#### Single-cell sorting of antigen-specific B cells

Human B cells were enriched from cryopreserved PBMCs using the EasySep™ Human B Cell Enrichment Kit (STEMCELL Technologies) according to the manufacturer’s instructions. After purification, B cells were stained with Zombie Aqua™ Fixable Viability Kit (Biolegend) and blocked with FcR Blocking Reagent (Miltenyi Biotec) for 15 min on ice. Following the 15 min incubation, B cells were washed with FACS buffer (phosphate-buffered saline (PBS) + 2% FBS +2 mM EDTA) and incubated with biotinylated SARS-CoV-2 RBD protein for 60 min at on ice. His-tag labeled SARS-CoV-2 RBD (The Native Antigen Company) was biotinylated with the EZ-Link Sulfo-NHS-LC-Biotin kit (Thermofisher Scientific) according to the manufacturer’s protocol, corresponding to 1-3 biotin groups per antibody molecule. After the 60 min incubation, B cells were washed with FACS buffer and stained with PerCP-cy5.5 anti-human CD19 antibody (Biolegend, 363016), FITC anti-human CD3 antibody (Biolegend, 300306) and PE streptavidin (Biolegend, 405203) for 25 min on ice. Subsequently, the stained cells were washed twice with FACS buffer and single-cell sorted by BD Influx™ Cell Sorter (BD Biosciences). UltraComp eBeads™ Compensation Beads and tosylactivated M−280 Dynabeads (Invitrogen) coupled with SARS-CoV-2 RBD protein according to the manufacturer’s instructions were used for compensation. Selection of RBD-specific B cells was performed using the following gating strategy: lymphocytes were selected using FSC and SSC, followed by selection of single cells based on width versus area of FSC and SSC signals. Next, live cells were selected as Zombia Aqua negative cells. Here, B cells were selected as CD19^+^ CD3^−^cells and evaluated for RBD surface staining. To allow proper gating of RBD-positive cells, a fluorescence minus one (FMO) control was included (Figure S1). Individual B cells were sorted into 96-wells PCR plates (Bioké) containing 2 μL lysis buffer (0.2% (v/v) Triton X-100 + 2U/μL RNaseOUT™ Recombinant Ribonuclease (Invitrogen) in UltraPure™ DNAse/RNase free distilled water (Invitrogen)) per well. The plates were sealed with a Microseal® ‘F’ Film (BioRad) and immediately frozen on dry ice before storage at −80°C for further use.

#### Amplification of antibody variable domains

Transcripts of lysed single B cells were denatured and hybridized with a mix of 1 μL 10 mM each nucleotide dNTP-Mix (Invitrogen) and 1 μL 10 μM oligo-dT primer (5′–AAGCAGTGGTATCAACGCAGAGTACT_30_-3′) at 72°C for 3 min. Subsequently, cDNA synthesis and pre-amplification was performed according to Picelli et al. (Picelli et al., 2014). cDNA was stored at −20°C. cDNA was purified with Agencourt AMPure XP beads (Beckman Coulter) according to the manufacturer’s protocol and quantified on the BioAnalyzer 2100 instrument (Agilent) and on a Qubit™ fluorometer (Thermofisher Scientific). The sequences encoding the immunoglobulin (Ig) heavy- and light-chain variable regions (V_H_ and V_L_) of IgM and IgG were amplified using reverse primers described by Ozawa et al. (Ozawa et al., 2006) and forward primer described by Picelli et al. (2014). The PCR reaction was performed with KAPA HiFi HotStart ReadyMix (Roche) at 95°C for 3 min, followed by 30 cycles at 98°C for 20 s, 60°C for 45 s and 72°C for 60 s and terminated at 72°C for 5 min. All PCR products were checked on a 1% agarose gel and purified using the Agencourt AMPure XP beads. Concentrations were measured on a Qubit™ fluorometer for Sanger sequencing (outsourced).

#### Recombinant antibody production and purification

##### Generation and amplification of expression vectors containing the selected heavy- and light chain sequences

Generation of antibody expression vectors was outsourced (Genscript). The Ig V_H_ and V_L_ sequences were cloned into a pcDNA3.4 expression vector containing human IgG1 constant regions. The plasmid DNA (pDNA) was amplified by transformation in DH5α *E. coli* and subsequent purification using the NucleoBond Xtra Maxi EF kit (Machery - Nagel) according to the manufacturer’s instructions. Plasmid purity and integrity was checked on a 1% agarose gel.

##### Production of recombinant antibody in HEK293-F cells

Recombinant antibodies were transiently transfected and produced *in vitro* in HEK293-F Freestyle suspension cells (Thermofisher Scientific). Briefly, equal amounts of heavy- and light chain pDNA were mixed with X-tremeGENE HP DNA Transfection Reagent (Roche) in FreeStyle 293 Expression Medium (Thermofisher Scientific) according to the manufacturer’s protocol and incubated for at least 15 min to allow the pDNA to enter the liposomes. The transfection reagent mixture was added in a dropwise manner to 293F Freestyle suspension cells. The cells were cultured in T175 flasks (Sarstedt, Germany) on an orbital shaker (Thermofischer Scientific) at 135 rpm and 8% CO_2_ in a 37°C humidified incubator for 5 days. At day 5, supernatant containing the recombinant antibodies was collected by centrifugation (1,400 x g for 10 min at room temperature), filtered through a 0.2 μm filter unit (VWR) and stored at −20°C.

##### Purification of recombinant antibody

Purification of the recombinant antibodies was conducted on a ÄKTAprime plus system (GE Healthcare Life Sciences) at 4°C using a 1 mL HiTrap® Protein A HP pre-packed Protein A Sepharose® column (Cytiva). Briefly, the column was first equilibrated in buffer A (20 mM sodium phosphate, 150mM NaCl, pH 7.5) before the sample was loaded on the column. The flow rate for all steps was 1 mL/min. The column was washed with buffer A and buffer B (20 mM Sodium Phosphate, 500 mM NaCl, pH 7.5). The recombinant antibodies were eluted from the column with 100 mM sodium acetate, pH 3.5 and the collected fractions were immediately neutralized with 1M Tris, pH 9. The fractions were pooled and dialyzed against sterile-filtered PBS. The ÄKTAprime plus system and all glasswork was treated with 30% hydrogen peroxide (VWR) to reduce the presence of endotoxin.

##### Quality control using SDS-PAGE and endotoxin measurements

Purified recombinant antibodies were assessed by SDS-PAGE under non-reducing conditions using the Amersham Phastsystem™ (GE Healthcare) according to the manufacturer’s instructions. Endotoxin levels were measured on Endosafe®-PTS™ (Charles River) according to the manufacturer’s instructions.

#### ELISA

##### Antigen binding

The binding of the purified recombinant antibodies to the following SARS-CoV-2 antigens was assessed via ELISA: spike glycoprotein (S1) RBD-His (REC31849-500, The Native Antigen Company), RBD(N439K)-His (40592-V08H14, Sino Biological), RBD(N501Y)-His (40592-V08H82, Sino Biological), RBD(E484K)-His (40592-V08H84, Sino Biological), RBD(Y453F)-His (40592-V08H80, Sino Biological), RBD(E484Q)-His (40592-V08H81, Sino Biological), RBD(L425R)-His (40592-V08H28, Sino Biological), RBD(L425R,E484Q)-His (40592-V08H88, Sino Biological), RBD(K417N, E484K, N501Y)-His (40592-V08H85, Sino Biological), Spike Glycoprotein (Full-Length)-His (REC31868-500, The Native Antigen Company). Briefly, polystyrene 96-well flat-bottom microtiter plates (Corning costar) were coated with 100 μL/well of 2 μg/mL antigen diluted in PBS and incubated overnight at 4°C. The next day, plates were blocked with 200 μL/well blocking buffer containing 1% (w/v) Bovine Serum Albumin (BSA, Sigma Aldrich) in PBS for 2 hours at room temperature. The plates were then washed six times with wash buffer (0.002% (v/v) Tween-80 (Sigma Aldrich) in PBS). After washing, 100 μL/well of the samples, calibrators and/or controls were added. As a positive and negative control, an anti-SARS-CoV-2 RBD mAb (40150-D004, Sino Biological) and anti-SARS-CoV-2 nucleocapsid antibody (MBS2563841, MyBioSource) were used, respectively. All samples and controls were diluted in PBS + 0.1% (w/v) BSA + 0.002% (v/v) Tween 80 with or without 1,86 g/L EDTA (PTA(E) buffer). After incubation, the plates were washed six times and incubated with 100 μL/well horseradish peroxidase (HRP)-conjugated goat anti-human IgG (Fc-specific, Sigma Aldrich) diluted 1/5000 in PTA buffer. After 1 h incubation at room temperature, the plate was again washed six times and 100 μL/well substrate (200 μL 40 mg/mL o-Phenylenediamine dihydrochloride 99+% (OPD, Acros Organics BVBA) + 2 μL H_2_O_2_ (Merck) in 20 mL citrate buffer, pH 5 (0.1 M citric acid monohydrate from Sigma and 0.2 M disodium phosphate dihydrate from Merck)) was added to the plate and incubated for 30 min in the dark. The color reaction was stopped with 50 μL/well 4 M H_2_SO_4_ (Thermofisher Scientific). Optical density (OD) was measured at 492 nm with an ELx808 ELISA reader (BioTek). Assay detection limits were determined based on the lowest sample dilution in combination with the OD and calculated concentration of the lowest point of the calibration curve. Analysis was performed using Graphpad Prism 9.0 (Graphpad Software).

##### Epitope binning assay

Polystyrene 96-well flat-bottom microtiter plates were coated with 4 μg/mL purified recombinant capture antibody in PBS (100 μL/well) and incubated at 4°C. After an overnight incubation, plates were blocked with 200 μL/well blocking buffer for 2 h at room temperature. The plates were washed six times with washing buffer and 10 ng/mL SARS-CoV-2 RBD protein diluted in PTA buffer (100 μL/well) was added to the plates for a 2-h incubation at room temperature. After the incubation, the plates were washed and captured antigen was detected with 100 μL/well of 1/100 biotin-conjugated purified recombinant antibodies diluted in PTA buffer. Biotinylation of the purified recombinant mAbs was performed with the EZ-Link Sulfo-NHS-LC-Biotin kit according to the manufacturer’s protocol. After a 1-h incubation at room temperature and washing, biotinylated recombinant antibodies that were able to bind the antigen were detected with 1/10,000 poly-HRP-conjugated streptavidin (Sanquin) diluted in PTA buffer (100 μL/well) and incubated for an additional 30 min at room temperature. After a final washing step, 100 μL/well substrate was added to the plate and incubated at room temperature for 30 min in the dark at room. The color reaction was stopped with 50 μL/well 4 M H_2_SO_4_. Optical density (OD) was measured at 492 nm with an ELx808 ELISA reader (BioTek). Very low OD values indicate the detection antibody was not able to bind, suggesting similar epitopes of both coating and detection antibodies, while high OD values indicate both antibodies have a different epitope. Epitope binning graphs were made in Microsoft Excel and clustering was done with ClustVis web tool (https://biit.cs.ut.ee/clustvis/).

##### Surface plasmon resonance

Surface Plasmon Resonance (SPR) was used to evaluate the interaction between mAbs and SARS-CoV-2 antigens (i.e. full-length spike protein, RBD and RBD mutants; see catalog numbers under Methods section “ELISA”). The binding experiments were performed at 25°C on a Biacore T200 instrument (GE Healthcare, Uppsala, Sweden) in HBS-EP+ buffer (10 mM HEPES, 150 mM NaCl, 3 mM EDTA and 0.05% v/v Surfactant P20). First, mouse anti-human IgG (Fc) antibody (Human Antibody Capture Kit, Cytiva) was immobilized on a CM5 chip according to manufacturer instructions. mAbs were then captured between 30 and 100 RU. Increasing concentrations of analyte were sequentially injected in one single cycle at a flow rate of 30 μl/min. The dissociation was monitored for 30 min. The chip was finally regenerated with 3M MgCl2 before a new mAb was captured. A reference flow was used as a control for non-specific binding and refractive index changes. Several buffer blanks were used for double referencing. Binding affinities (KD) were derived after fitting the experimental data to the 1:1 binding model in the Biacore T200 Evaluation Software 3.1 using the single cycle kinetic procedure. Each interaction was repeated a least three times.

### Monoclonal antibody neutralization assays: production of S-pseudotyped virus and serum neutralization test

VSV S-pseudotypes were generated as described previously (Sanchez-Felipe et al., 2021). Briefly, depending on the plasmid backbone, BHK-21J cells (D614G (Sanchez-Felipe et al., 2021) and omicron (Sharma et al., 2022), cloned into pCAGGS) or HEK-293-T cells (beta, gamma and delta, as sourced from Invivogen Cat. No. plv-spike-v3, plv-spike-v5 and plv-spike-v8, respectively) were transfected with the respective S protein expression plasmids, and one day later infected with GFP-encoding VSVΔG backbone virus. Two hours later, the medium was replaced by medium containing anti-VSV-G antibody (I1-hybridoma, ATCC CRL-2700) to neutralize residual VSV-G input. After 26 h incubation at 32°C, the supernatants were harvested. To quantify nAbs, serial dilutions of serum samples were incubated for 1 h at 37°C with an equal volume of S pseudotyped VSV particles and inoculated on Vero E6 cells for 19 h. The percentage of GFP expressing cells was quantified on a Cell Insight CX5/7 High Content Screening platform (Thermo Fisher Scientific) with Thermo Fisher Scientific HCS Studio (v.6.6.0) software.

#### Golden syrian hamster studies

##### SARS-CoV-2 infection model in hamsters

The hamster infection model of SARS-CoV-2 has been described before (Boudewijns et al., 2020; Kaptein et al., 2020). For infection, animals were anesthetized with ketamine/xylazine/atropine and inoculated intranasally with 50 μL containing either 2×10^6^ TCID50 SARS-CoV-2 (ancestral Wuhan strain), 1×10^4^ TCID50 (Beta B.1.351), or 1×10^4^ TCID50 (Delta B.1.617.2). Antibody protein treatments (anti-SARS-CoV-2 mAbs or human IgG1 isotype control Trastuzumab/Herceptin® (Roche)) were initiated 24 h post infection by intraperitoneal injection. Intramuscular pDNA electroporation was done at d-10, d-7 and d-5 infection. Hamsters were monitored for appearance, behavior, and weight. At day 4 post-infection, animals were euthanized by intraperitoneal injection of 500 μL Dolethal (200 mg/mL sodium pentobarbital, Vétoquinol SA). Lungs were collected and viral RNA and infectious virus were quantified by RT-qPCR and end-point virus titration, respectively. Blood samples were collected at end-point for pharmacokinetic analysis. No randomization methods were used and confounders were not controlled, though all caretakers and technicians were blinded to group allocation in the animal facility and to sample numbers for analysis (qPCR, titration, and histology).

##### Intramuscular pDNA electroporation

3B8 was delivered *in vivo,* encoded in the CMV-driven pcDNA3.4 vectors, as an equimolar mixture of the 3B8 heavy and light chain plasmids (jointly referred to as ‘p3B8’). Hamsters received an intramuscular p3B8 injection in their left and right tibialis anterior and gastrocnemius muscle (pretreated with hyaluronidase), followed by electroporation. The procedure was performed by adapting a previously optimized and validated pre-clinical protocol for mice (Hollevoet et al., 2018). In brief, fur at the target sites was removed using depilatory product (Veet, Reckitt Benckiser), at least two days prior to pDNA injection. Intramuscular delivery sites were injected with 100 μL of 0.4 U/μl hyaluronidase from bovine testes reconstituted in sterile saline (H4272, Sigma-Aldrich), approximately 1 h prior to pDNA electrotransfer. Total p3B8 amount delivered per hamster was 600 μg (75 μL pDNA, at 2 μg/μL per muscle, formulated in sterile MQ H2O). Intramuscular injections of pDNA were immediately followed by *in situ* electroporation using the NEPA21 Electroporator (Sonidel) with CUY650P5 tweezer electrodes at a fixed width of 7 mm. Signa Electrode Gel (Parker Laboratories) was applied to the muscle to target an impedance below 0.6 Ohm. Three series of four 20 ms square-wave pulses of 120 V with a 50 ms interval were applied with polarity switching after two of the four pulses. During the procedures, hamsters were sedated using isoflurane inhalation. Electroporation parameters were based on pilot studies in hamster with a firefly luciferase reporter pDNA (data not shown). Pulse delivery was verified using the NEPA21 readout.

##### SARS-CoV-2 RT-qPCR

Hamster lung tissues were collected after sacrifice and were homogenized using bead disruption (Precellys) in 350 μL TRK lysis buffer (E.Z.N.A. Total RNA Kit, Omega Bio-tek) and centrifuged (10.000 rpm, 5 min) to pellet the cell debris. RNA was extracted according to the manufacturer’s instructions. RT-qPCR was performed on a LightCycler96 platform (Roche) using the iTaq Universal Probes One-Step RT-qPCR kit (BioRad) with N2 primers and probes targeting the nucleocapsid (Boudewijns et al., 2020). Standards of SARS-CoV-2 cDNA (IDT) were used to express viral genome copies per mg tissue (Kaptein et al., 2020).

##### Endpoint virus titrations

Lung tissues were homogenized using bead disruption (Precellys) in 350 μL minimal essential medium and centrifuged (10,000 rpm, 5min, 4°C) to pellet the cell debris. To quantify infectious SARS-CoV-2 particles, endpoint titrations were performed on confluent Vero E6 cells in 96- well plates. Viral titers were calculated by the Reed and Muench method (Reed and Muench, 1938) using the Lindenbach calculator and were expressed as 50% tissue culture infectious dose (TCID_50_) per mg tissue.

##### Histology

For histological examination, the lungs were fixed overnight in 4% formaldehyde and embedded in paraffin. Tissue sections (5 μm) were analyzed after staining with hematoxylin and eosin and scored blindly for lung damage by an expert pathologist. The scored parameters, to which a cumulative score of 1 to 10 was attributed, were the following: congestion, intra-alveolar hemorrhagic, intra-alveolar edema, apoptotic bodies in bronchus wall, necrotizing bronchiolitis, perivascular edema, bronchopneumonia, perivascular inflammation, peribronchial inflammation and vasculitis. Representative histology images used to generate cumulative scores are shown in Figures S2 and S3.

### Quantification and statistical analysis

All statistical analyses were performed using GraphPad Prism 9 software (GraphPad Software, Inc.). Neutralization IC50 values were determined by normalizing the serum neutralization dilution curve to a virus (100%) and cell control (0%) and fitting in Graphpad Prism. Statistical significance between two treatment groups was determined using Mann Whitney U-test. P-values of <0.05 were considered significant. Statistical details of the experiments can be found in the respective figure legends.

#### Hamster studies

##### Sample size justification

For antiviral efficacy, we want to detect at least 1 log_10_ reduction in viral RNA levels in treated subjects compared to the untreated, infected control group. Group size was calculated based on the independent t-test with an effect size of 2.0 and a power of 80% (effect size = delta mean/SD = 1 log_10_ decrease in viral RNA/0.5 log_10_), resulting in 5-6 animals/group. Sample sizes maximized considering limits in BSL3 housing capacity, numbers of animals that can be handled under BSL3 conditions, and availability of compounds.

##### Data exclusion

In the hamster studies performed in this project, mAb treatments were injected intraperitoneally to evaluate their therapeutic potential. A common problem with IP injection is that the treatment is sometimes deposited (either fully or partially) into unintended sites such as the abdominal fat or subcutaneous tissues (misinjection). To determine in which animals misinjection of the therapeutic mAb had occurred, we analyzed the mAb serum concentrations by ELISA three days after ip injection (i.e. day 4 of the experiment). If no mAb could be detected in the serum, these animals were considered not efficiently injected and excluded from further analysis. This is also described in the results section of the respective experiments.

## Data Availability

• The data reported in this paper will be shared by the lead contact upon request. • This paper does not report original code. • Any additional information required to reanalyze the data reported in this paper is available from the lead contact upon request

## References

[bib2] Abdelnabi R., Boudewijns R., Foo C.S., Seldeslachts L., Sanchez-Felipe L., Zhang X., Delang L., Maes P., Kaptein S.J.F. (2021). Comparing infectivity and virulence of emerging SARS-CoV-2 variants in Syrian hamsters. EBioMedicine.

[bib3] Andreano E., Andreano E., Nicastri Emanuele, Paciello Ida, Pileri Piero, Manganaro Noemi, Piccini Giulia, Manenti Alessandro, Pantano Elisa, Kabanova Anna, Troisi Marco, Vacca Fabiola (2021). Extremely potent human monoclonal antibodies from COVID-19 convalescent patients’. Cell.

[bib4] Andrews C.D., Huang Y., Ho D.D., Liberatore R.A. (2020). In vivo expressed biologics for infectious disease prophylaxis: rapid delivery of DNA-based antiviral antibodies. Emerg. Microbes Infect..

[bib5] Andrews N., Tessier Elise, Stowe Julia, Gower Charlotte, Kirsebom Freja, Simmons Ruth, Gallagher Eileen, Chand Meera, Brown Kevin, Ladhani Shamez N. (2021). Vaccine effectiveness and duration of protection of comirnaty, vaxzevria and Spikevax against mild and severe COVID-19 in the UK. medRxiv.

[bib6] Barnes C.O., Jette C.A., Abernathy M.E., Dam K.A., Esswein S.R., Gristick H.B., Malyutin A.G., Sharaf N.G., Huey-Tubman K.E., Lee YE (2020). SARS-CoV-2 neutralizing antibody structures inform therapeutic strategies. Nature.

[bib7] Baum A., Fulton B.O., Wloga E., Copin R., Pascal K.E., Russo V., Giordano S., Lanza K., Negron N., Ni M. (2020). Antibody cocktail to SARS-CoV-2 spike protein prevents rapid mutational escape seen with individual antibodies. Science.

[bib8] Baum A., Ajithdoss D., Copin R., Zhou A., Lanza K., Negron N., Ni M., Wei Y., Mohammadi K., Musser B. (2020). REGN-COV2 antibodies prevent and treat SARS-CoV-2 infection in rhesus macaques and hamsters. Science.

[bib9] Boudewijns R., Thibaut H.J., Kaptein S.J.F., Li R., Vergote V., Seldeslachts L., Van Weyenbergh J., De Keyzer C., Bervoets L., Sharma S. (2020). STAT2 signaling restricts viral dissemination but drives severe pneumonia in SARS-CoV-2 infected hamsters. Nat. Commun..

[bib10] Calcoen, B. et al. (no date) Real-world monitoring of BNT162b2 vaccine-induced SARS-CoV-2 B and T cell immunity in naive healthcare workers: a prospective single center study [manuscript in preparation].

[bib11] Cameroni E., Bowen J.E., Rosen L.E., Saliba C., Zepeda S.K., Culap K., Pinto D., VanBlargan L.A., De Marco A., di Iulio J. (2022). Broadly neutralizing antibodies overcome SARS-CoV-2 Omicron antigenic shift. Nature.

[bib12] CDC (2021). SARS-CoV-2 variant classifications and definitions. https://www.cdc.gov/coronavirus/2019-ncov/variants/variant-info.html#Interest.

[bib13] Chen J., Gao K., Wang R., Wei G.W. (2021). Revealing the threat of emerging SARS-CoV-2 mutations to antibody therapies. J. Mol. Biol..

[bib14] Chen R.E., Zhang X., Case J.B., Winkler E.S., Liu Y., VanBlargan L.A., Liu J., Errico J.M., Xie X., Suryadevara N. (2021). Resistance of SARS-CoV-2 variants to neutralization by monoclonal and serum-derived polyclonal antibodies. Nat. Med..

[bib15] Cohn B.A., Cirillo Piera M., Murphy Caitlin C., Krigbaum Nickilou Y., Wallace Arthur W. (2021). Breakthrough SARS-CoV-2 infections in 620,000 U.S. Veterans, february 1, 2021 to August 13. medRxiv.

[bib16] Collier D.A., De Marco A., Ferreira I.A.T.M., Meng B., Datir R.P., Walls A.C., Kemp S.A., Bassi J., Pinto D., Silacci-Fregni C. (2021). Sensitivity of SARS-CoV-2 B.1.1.7 to mRNA vaccine-elicited antibodies. Nature.

[bib17] Connor B.A., Couto-Rodriguez M., Couto-Rodriguez M., Barrows J.E., Gardner M., Rogova M. (2021). Monoclonal antibody therapy in a vaccine breakthrough SARS-CoV-2 hospitalized delta (B.1.617.2) variant case. Int. J. Infect. Dis..

[bib18] Corti D., Purcell L.A., Snell G., Veesler D. (2021). Tackling COVID-19 with neutralizing monoclonal antibodies. Cell.

[bib19] European Medicines Agency (2022). https://www.ema.europa.eu/en/human-regulatory/overview/public-health-threats/coronavirus-disease-covid-19/treatments-vaccines/covid-19-treatments.

[bib20] Fagre A.C., Manhard J., Adams R., Eckley M., Zhan S., Lewis J., Rocha S.M., Woods C., Kuo K., Liao W. (2020). A potent SARS-CoV-2 neutralizing human monoclonal antibody that reduces viral burden and disease severity in Syrian hamsters. Front. Immunol..

[bib21] Food and Drug Administration (2021).

[bib22] Goldberg Y., Mandel M., Bodenheimer O., Bar-On Y.M. (2021). Waning immunity of the BNT162b2 vaccine: A Nationwide Study from Israel. medRxiv.

[bib23] Greaney A.J., Starr T.N., Barnes C.O., Weisblum Y., Schmidt F., Caskey M., Gaebler C. (2021). Mapping mutations to the SARS-CoV-2 RBD that escape binding by different classes of antibodies. Nature Communications.

[bib24] Hacisuleyman E., Hale C., Saito Y., Blachere N.E., Bergh M., Conlon E.G., Schaefer-Babajew D.J., DaSilva J., Muecksch F., Gaebler C. (2021). Vaccine breakthrough infections with SARS-CoV-2 variants. N. Engl. J. Med..

[bib25] Hansen J., Baum A., Pascal K.E., Russo V., Giordano S., Wloga E., Fulton B.O., Yan Y., Koon K., Patel K. (2020). Studies in humanized mice and convalescent humans yield a SARS-CoV-2 antibody cocktail. Science.

[bib26] Hoffmann M., Arora P., Groß R., Seidel A., Hörnich B.F., Hahn A.S., Krüger N., Graichen L., Hofmann-Winkler H., Kempf A. (2021). SARS-CoV-2 variants B.1.351 and P.1 escape from neutralizing antibodies. Cell.

[bib27] Hollevoet K., De Smidt E., Geukens N., Declerck P. (2018). Prolonged in vivo expression and anti-tumor response of DNAbased anti-HER2 antibodies. Oncotarget.

[bib28] Hollevoet K., De Vleeschauwer S., De Smidt E., Vermeire G., Geukens N., Declerck P. (2019). Bridging the clinical gap for DNA-Based antibody therapy through translational studies in sheep. Hum. Gene Ther..

[bib29] Hollevoet K., Declerck P.J. (2017). State of play and clinical prospects of antibody gene transfer’, *Journal of Translational Medicine*. BioMed Central.

[bib30] Jones B.E., Brown-Augsburger P.L., Corbett K.S., Westendorf K., Davies J. (2020). LY-CoV555, a rapidly isolated potent neutralizing antibody, provides protection in a non-human primate model of SARS-CoV-2 infection. bioRxiv.

[bib31] Ju B., Zheng Qingbing, Guo Huimin, Fan Qing, Li Tingting, Song Shuo, Sun Hui, Shen Senlin, Zhou Xinrong, Cheng Lin, Xue Wenhui (2022). Molecular basis of broad neutralization against SARS-CoV-2 variants including Omicron by a human antibody. bioRxiv.

[bib32] Kaptein S.J.F., Jacobs S., Langendries L., Seldeslachts L., Seldeslachts L., Liesenborghs L., Hens B., Vergote V., Heylen E., Barthelemy K. (2020). Favipiravir at high doses has potent antiviral activity in SARS-CoV-2−infected hamsters, whereas hydroxychloroquine lacks activity. Proc. Natl. Acad. of Sci. USA.

[bib33] Kreye J., Momsen Reincke S., Kornau Hans-Christian, Sánchez-Sendin Elisa, Corman Victor Max, Liu Hejun, Yuan Meng (2020). A therapeutic non-self-reactive SARS-CoV-2 antibody protects from lung Pathology in a COVID-19 hamster model. Cell.

[bib34] Kustin T., Harel Noam, Finkel Uriah, Perchik Shay, Harari Sheri, Tahor Maayan, Caspi Itamar, Levy Rachel, Leschinsky Michael, Ken Dror Shifra (2021). Evidence for increased breakthrough rates of SARS-CoV-2 variants of concern in BNT162b2 mRNA vaccinated individuals. medRxiv.

[bib35] Lassaunière R., Fonager J., Rasmussen M., Frische A.,M., Frische A., Rasmussen T.B. (2021). In vitro characterization of fitness and convalescent antibody neutralization of SARS-CoV-2 cluster 5 variant emerging in mink at Danish farms. Front. Microbiol..

[bib36] Lee W.S., Wheatley A.K., Kent S.J., DeKosky B.J. (2020). Antibody-dependent enhancement and SARS-CoV-2 vaccines and therapies. Nature Microbiology.

[bib37] Li M., Lou F., Fan H. (2022). SARS-CoV-2 variant Omicron: currently the most complete “escapee” from neutralization by antibodies and vaccines. Signal Transduct. and Target. Ther..

[bib38] Lindenbach B.D., Rice C.M. (1997). trans-Complementation of yellow fever virus NS1 reveals a role in early RNA replication. J. Virol..

[bib39] McCallum M., De Marco A., Chen A., Bassi J., Walls A.C., Di Iulio J., Di Iulio J. (2021). SARS-CoV-2 immune evasion by variant B.1.427/B.1.429. bioRxiv.

[bib40] Ozawa T., Kishi H., Muraguchi A. (2006). Amplification and analysis of cDNA generated from a single cell by 5′-RACE: application to isolation of antibody heavy and light chain variable gene sequences from single B cells. BioTechniques.

[bib41] Picelli S., Faridani O.R., Björklund A.K., Winberg G., Sagasser S., Sandberg R. (2014). Full-length RNA-seq from single cells using Smart-seq2. Nat. Protoc..

[bib42] Piepenbrink M.S. (2021). Therapeutic activity of an inhaled potent SARS-CoV-2 neutralizing human monoclonal antibody in hamsters. Cell Reports Medicine.

[bib43] Planas D., Veyer D., Baidaliuk A., Staropoli I., Guivel-Benhassine F., Rajah M.M., Planchais C., Porrot F., Robillard N., Puech J. (2021). Reduced sensitivity of SARS-CoV-2 variant Delta to antibody neutralization. Nature.

[bib44] Reed L., Muench H. (1938). A simple method of estimating fifty per cent endpoints. Am. J. Epidemiol..

[bib45] Ritchie H. (2021). Coronavirus pandemic (COVID-19). https://ourworldindata.org/coronavirus.

[bib46] Ryu D.-K., Song R. (2021). Therapeutic effect of CT-P59 against SARS-CoV-2 South African variant Dong-Kyun. bioRxiv.

[bib47] Ryu D.-K., Kang B. (2021). Therapeutic efficacy of CT-P59 against P.1 variant of SARS-CoV-2. bioRxiv.

[bib48] Ryu D. (2021). The in vitro and in vivo potency of CT-P59 against Delta and its associated variants of SARS-CoV-2 Dong-Kyun Ryu. bioRxiv.

[bib49] Sanchez-Felipe L., Vercruysse T., Sharma S., Ma J., Lemmens V., Van Looveren D., Arkalagud Javarappa M.P., Boudewijns R., Malengier-Devlies B., Liesenborghs L. (2021). A single-dose live-attenuated YF17D-vectored SARS-CoV-2 vaccine candidate. Nature.

[bib50] Sanyaolu A. (2021). The emerging SARS-CoV-2 variants of concern. Therapeutic Advances in Vaccines.

[bib51] Sharma S., Vercruysse T., Sanchez-Felipe L., Kerstens W., Rasulova M., Abdelnabi R., Foo C.S., Lemmens V., Van Looveren D., Maes P. (2022). Updated vaccine protects from infection with SARS-CoV-2 variants, prevents transmission and is immunogenic against Omicron in hamsters. bioRxiv.

[bib52] Tartof S.Y. (2021). Effectiveness of mRNA BNT162b2 COVID-19 vaccine up to 6 months in a large integrated health system in the USA: a retrospective cohort study. Lancet.

[bib53] Taylor P.C. (2021). Neutralizing monoclonal antibodies for treatment of COVID-19. Nat. Rev. Immunol..

[bib54] Thomson E.C., Rosen L.E., Shepherd J.G., Spreafico R., da Silva Filipe A., Wojcechowskyj J.A., Davis C., Piccoli L., Pascall D.J., Dillen J. (2021). Circulating SARS-CoV-2 spike N439K variants maintain fitness while evading antibody-mediated immunity. Cell.

[bib55] Tortorici M.A., Beltramello M., Lempp F.A., Pinto D., Dang H.V., Rosen L.E., McCallum M., Bowen J., Minola A., Jaconi S. (2020). Ultrapotent human antibodies protect against SARS-CoV-2 challenge via multiple mechanisms. Science.

[bib56] Touret F. (2022). In Vitro Evaluation of Therapeutic Antibodies against a SARS-CoV-2 Short Communication. bioRxiv.

[bib57] Vermeire G., De Smidt E., Geukens N., Williams J.A., Declerck P., Hollevoet K. (2021). Improved potency and safety of DNA-encoded antibody therapeutics through plasmid backbone and expression cassette engineering. Hum. Gene Ther..

[bib58] Walls A.C., Young-Jun Park M., Alexandra Tortorici, Wall Abigail, Andrew T., David Veesler M.c.Guire (2020). Structure, function, and Antigenicity of the SARS-CoV-2 spike glycoprotein. Cell.

[bib59] Wang P., Nair M.S., Liu L., Iketani F., Iketani S., Guo Y., Wang M. (2021). Antibody resistance of SARS-CoV-2 variants B.1.351 and B.1.1.7’. Nature.

[bib60] Wang Z., Schmidt F., Weisblum Y., Muecksch F., Barnes C.O., Finkin S., Schaefer-Babajew D. (2021). mRNA vaccine-elicited antibodies to SARS-CoV-2 and circulating variants. Nature.

[bib61] Westendorf K., Žentelis S., Wang L., Foster D., Vaillancourt P., Wiggin M. (2022). LY-CoV1404 (bebtelovimab) potently neutralizes SARS-CoV-2 variants. bioRxiv.

[bib62] Winkler E.S., Gilchuk P., Yu J., Bailey A.L., Chen R.E., Chong Z. (2021). Human neutralizing antibodies against SARS-CoV-2 require intact Fc effector functions for optimal therapeutic protection. Cell.

[bib63] Loganathan S.K., Schleicher K., Malik A., Quevedo R., Langille E., Teng K., Oh R.H., Rathod B., Tsai R., Samavarchi-Tehrani P. (2020). Cryo-EM structure of the 2019-nCoV spike in the prefusion conformation. Science.

[bib64] Zhou D., Dejnirattisai W., Supasa F., Liu C., Mentzer A.J. (2021). Evidence of escape of SARS-CoV-2 variant B.1.351 from natural and vaccine-induced sera’. Cell.

