# Supplementary material for: Potent neutralizing anti-SARS-CoV-2 human antibodies cure infection with SARS-CoV-2 variants in hamster model
Source: iScience. 2022 Jul 3;25(8):104705. doi: 10.1016/j.isci.2022.104705 (PMC9250818; doi:10.1016/j.isci.2022.104705)
Supplement: Document S1. Figures S1–S3 and Tables S1–S3 [file mmc1.pdf]

iScience, Volume *25*

Supplemental information

Potent neutralizing anti-SARS-CoV-2 human antibodies cure infection with SARS-CoV-2 variants in hamster model

Maya Imbrechts, Wim Maes, Louanne Ampofo, Nathalie Van den Berghe, Bas Calcoen, Dominique Van Looveren, Winnie Kerstens, Madina Rasulova, Thomas Vercruysse, Sam Noppen, Rana Abdelnabi, Caroline Foo, Kevin Hollevoet, Piet Maes, Xin Zhang, Dirk Jochmans, Karen Ven, Jeroen Lammertyn, Karen Vanhoorelbeke, Nico Callewaert, Paul De Munter, Dominique Schols, Hendrik Jan Thibaut, Johan Neyts, Paul Declerck, and Nick Geukens

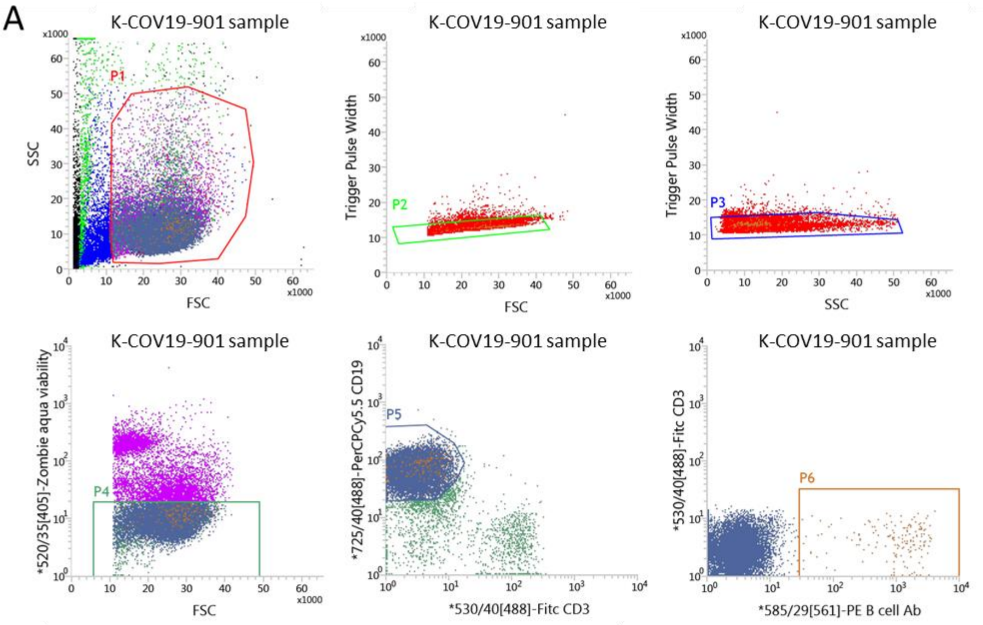

Flow cytometry gating strategy

Populations		Even...	% Total	% Parent
All Events		36,844	100.00%	####
P1	Lymphocytes	25,509	69.24%	69.24%
P2	Single cells	30,177	81.90%	81.90%
P3	Single cells	28,221	76.60%	76.60%
P1 AND P2 AND P3		24,595	66.75%	66.75%
P4	Live cells	18,562	50.38%	75.47%
P5	B cells	16,840	45.71%	90.72%
P6	RBD⁺ B cells	169	0.46%	1.00%

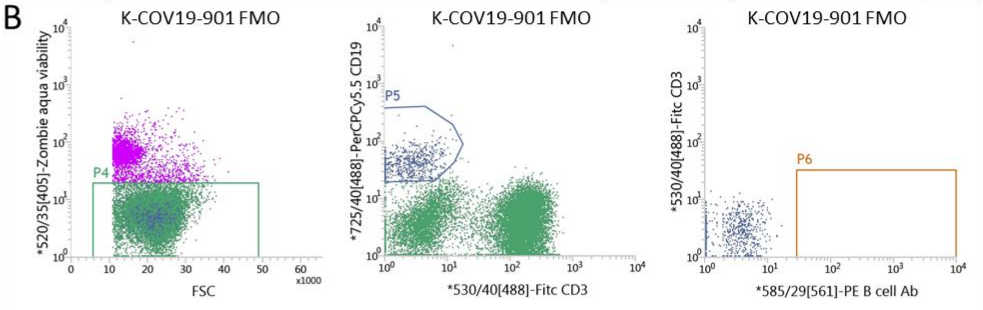

Figure S1, gating strategy for selection of RBD-specific B cells from PBMCs of patient K-COV19-901, related to STAR methods. Lymphocytes were selected based on FSC and SSC, followed by selection of single cells and live cells (Zombia Aqua negative). Here, B cells were selected as CD19+ CD3- cells and evaluated for RBD surface staining using PE as fluorophore. Fully stained sample is shown in panel A, fluorescence minus one (FMO) control for RBD fluorescence is shown in panel B.

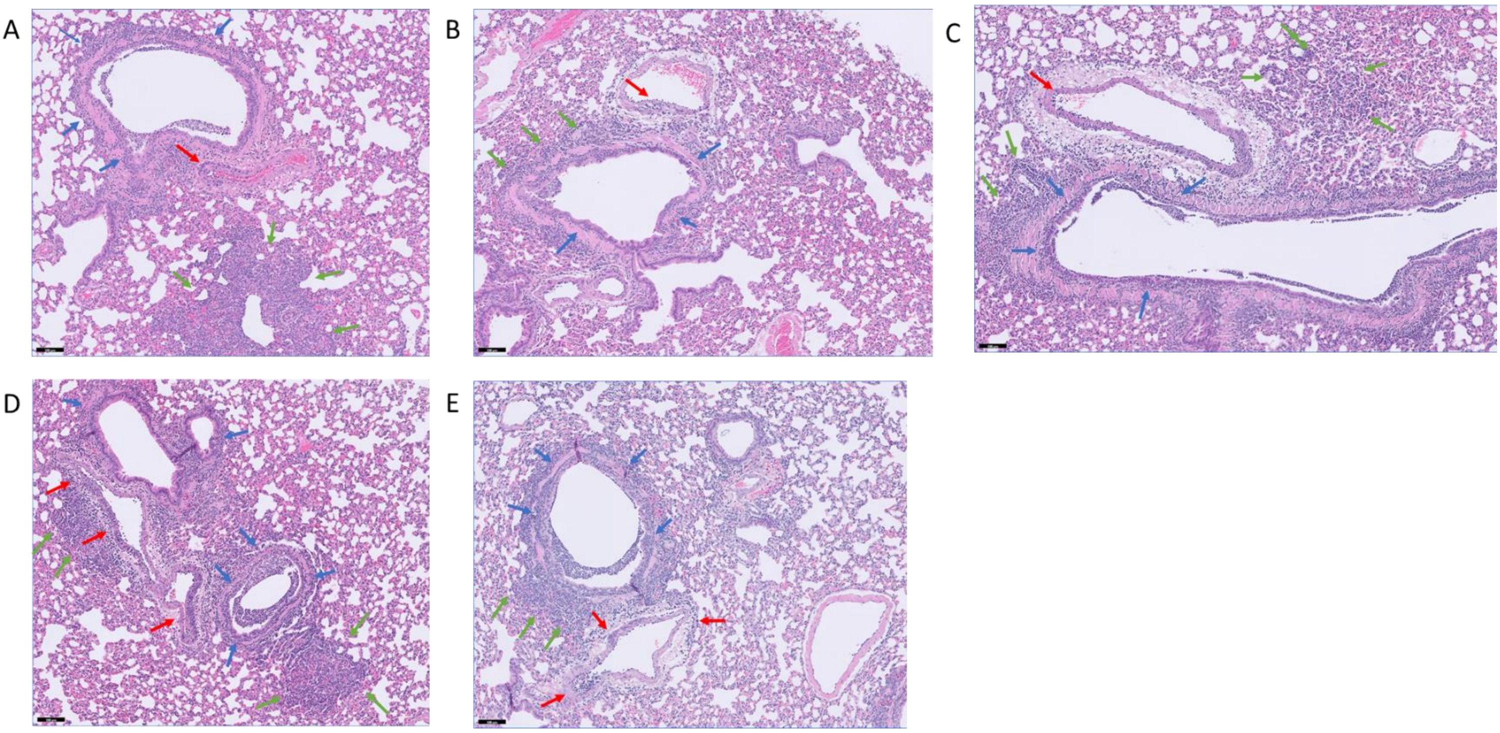

Figure S2, Representative histology images for treatment of SARS-CoV-2 delta with isotype, 3B8, 3E6, 2B2 or REGN-COV-2, related to Figure 5. A) Isotype control; perivascular inflammation with vasculitis (red arrows), peribronchial inflammation with intra-bronchial accumulation of neutrophils (blue arrows) and bronchopneumonia (green arrows). B) 3B8; perivascular inflammation with vasculitis (red arrow), limited peribronchial inflammation (blue arrows) and limited bronchopneumonia (green arrows). C) 3E6; perivascular inflammation with vasculitis (red arrow), limited peribronchial inflammation (blue arrows) and bronchopneumonia (green arrows). D) 2B2; perivascular inflammation with vasculitis (red arrows), peribronchial inflammation with intra-bronchial accumulation of neutrophils (blue arrows) and bronchopneumonia (green arrows). E) REGN-COV-2; perivascular inflammation with vasculitis (red arrows), peribronchial inflammation with intra-bronchial accumulation of neutrophils (blue arrows) and limited bronchopneumonia (green arrows). Scale bar size = 100 µm.

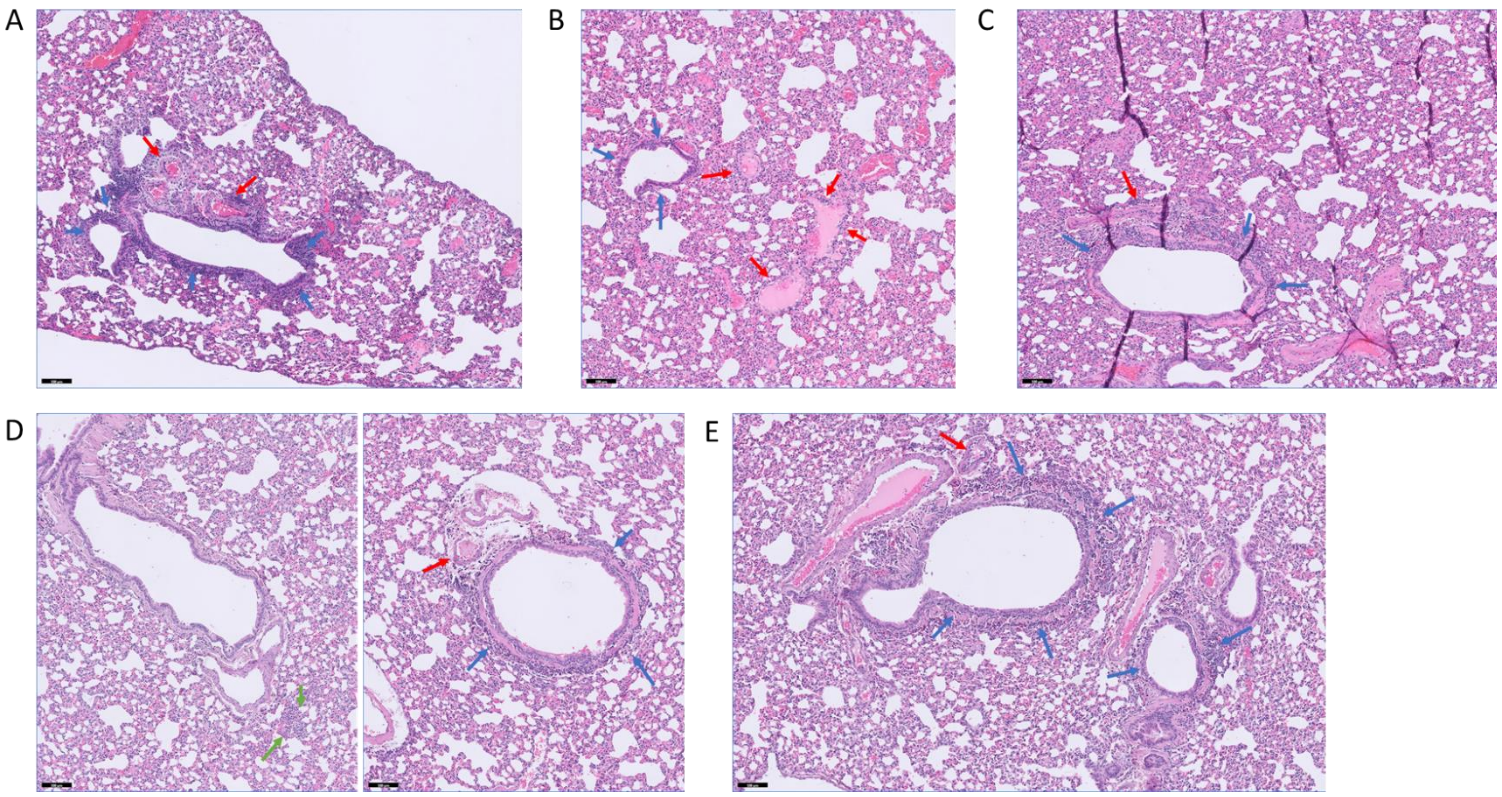

Figure S3, Representative histology images for dose-response treatment of SARS-CoV-2 delta with mAb 3B8 (5, 1, 0.2 or 0.04 mg/kg), related to Figure 6. A) Isotype control; limited peribronchial inflammation (blue arrows) and perivascular inflammation (red arrows). No vasculitis, intrabronchial neutrophils or bronchopneumonia. B) 3B8 5 mg/kg; very limited perivascular inflammation (red arrows). No peribronchial (blue arrows) or bronchopneumonia. C) 3B8 1 mg/kg; very limited peribronchial (blue arrows) and perivascular (red arrows) inflammation, no bronchopneumonia. D) 3B8 0.2 mg/kg; one microscopic focus of bronchopneumonia (green arrows). Limited perivascular inflammation (red arrow) and one bronchus with peribronchial inflammation (blue arrows). E) 3B8 0.04 mg/kg; very limited perivascular inflammation (red arrow). Peribronchial inflammation (blue arrows), but no bronchopneumonia. Scale bar size = 100 µm.

Table S1, COVID-19 patient characteristics, related to STAR methods. Overview of 25 included COVID-19 patients, their age and sex, RBD-binding titers and neutralizing antibody titers in serum, and percentage RBD-positive B cells in PBMCs.

Patient ID	Age	Sex	anti-RBD Ab (µg/ml)	Neutralizing Abs (SNT50 titer)	% RBD-positive of B cells
K-COV19-901	74	F	182,16	1209	0,80%
K-COV19-902	70	M	21,65	96	0,00%
K-COV19-006	66	M	387,59	7022	0,00%
K-COV19-007	62	M	5,98	/	0,00%
K-COV19-009	38	M	7,57	8	0,04%
K-COV19-010	27	F	15,58	74	0,03%
K-COV19-019	53	M	26,07	771	0,05%
K-COV19-021	66	M	30,80	506	0,19%
K-COV19-028	56	M	25,75	493	0,00%
K-COV19-034	61	M	48,82	810	0,02%
K-COV19-041	50	M	8,66	201	0,09%
K-COV19-044	64	F	40,22	876	0,00%
L-COV19-001	40	F	14,75	178	0,14%
L-COV19-002	54	M	24,37	248	0,30%
L-COV19-003	44	M	51,37	481	0,18%
L-COV19-004	53	M	48,71	302	0,07%
L-COV19-005	55	M	196,52	2123	0,14%
L-COV19-007	65	M	32,00	829	0,22%
L-COV19-008	80	M	54,97	1539	0,35%
L-COV19-009	81	M	32,07	1199	0,21%
L-COV19-010	58	M	16,76	104	0,19%
L-COV19-011	28	F	32,26	495	0,11%
L-COV19-012	56	F	13,99	140	0,07%
L-COV19-015	64	M	41,20	3077	0,14%
L-COV19-018	25	F	10,36	69	0,11%

Table S2, overview of single RBD mutants used in this study and their presence in different SARS-CoV-2 variants of concern, variants of interest, variants under monitoring or de-escalated variants, related to Table 2. Information retrieved from European Centre for Disease Prevention and Control (https://www.ecdc.europa.eu/en/covid-19/variants-concern)

	**SARS-CoV-2 variants**		
**Mutation**	**Concern**	**De-escalated***	**Other**
**N439K**		AV.1	Ab evasion mutant
**L452R**	B.1.617.2 (Delta)	C.37 (Lambda); B.1.427/B.1.429 (Epsilon); B.1.617.1 (Kappa); AY.4.2; B.1.617.3; A.27; C.16; B.1.526.1	
**Y453F**			Mink mutant, Ab evasion mutant
**E484K**	B.1.351 (beta); P.1 (Gamma)	B.1.621 (Mu); B.1.525 (Eta); P.3 (Theta); B.1.1.318; C.1.2; B.1.620; A.28; B.1.526 (Iota); P.2 (Zeta); AT.1; AV.1	
**E484Q**		B.1.617.1 (Kappa); B.1.617.3	
**N501Y**	B.1.351 (beta); P.1 (Gamma); B.1.1.529 (Omicron)	B.1.1.7 (Alpha); P.3 (Theta); B.1.621 (Mu); A.27	

**These variants of SARS-CoV-2 have been de-escalated based on at least one the following criteria: (1) the variant is no longer circulating, (2) the variant has been circulating for a long time without any impact on the overall epidemiological situation, (3) scientific evidence demonstrates that the variant is not associated with any concerning properties (https://www.ecdc.europa.eu/en/covid-19/variants-concern).*

Table S3, amino acid sequence of heavy chain and light chain variable domains of mAbs 3B8, 3E6, 2B2 and 1C1, related to Figures 3, 4, 5.

K-COV-901_3B8_HC	QVQLVQSGAEVKKPGASVKVSCKAYGYTFTSHYMHWVRQAPGQGLEWMGIIDSSGGGASYPQKFQGRVTLTRDTSSSTVYMELSSLRSEDTAVYYCAKAHSTNWYGWFDPWGQGTLVTVSS
K-COV-901_3B8_LC	QSVLTQPPSTSGTPGQRVTISCSGSSSNIGSNTVNWYQQLPGTAPKLLIYNNNQRPSGVPDRFSGSKSGTSASLAISGLQSEDEADYYCAAWDDGLNGSGWVFGGGTKLTVL
K-COV-901_3E6_HC	EVRLVESGGGLIQPGGSLTLSCAASGVIVSRNYMNWVRQAPGKGLEWVSVIYSGGSTFYADSVKGRFTISRDNSKNTLYLQMNSLRAEDTAVYYCARWGPNYYGSGSDHYNSYGMDVWGQGTTVTVSS
K-COV-901_3E6_LC	EIVLTQSPGTLSLSPGESATLSCRASQGVSSSSLAWYQQRPGQAPRLLMYGASSRATGIPDRFSGSGSGTDFTLTISRLEPEDFAVYYCQQSGSSPQYTFGQGTKLEIK
K-COV-901_2B2_HC	EVQLVESGGGLVQPGRSLRLSCAASGFTFDDYAMHWVRQAPGKGLEWVSGVSWNSGTIGYADSVKGRFIISRDNAKNSLYLQMRSLRAEDTALYYCAKDVSVYRFREQSYGMDVWGQGTTVTVLS
K-COV-901_2B2_LC	QSALTQSASVSGSPGQSITISCTGTSSDIGGYNFVSWYQQHPGKAPKLMIYEVTTRPSGVSNRFSGSKSGNTASLTISGLQAEDEADYYCSSYTSSSLVVFGGGTKLALL
K-COV-901_1C1_HC	EVQLVESGGGLVQPGRSLRLSCAASGFSLDDYGMHWVRQAPGKGLEWVSGISWNSGDRGYADSVKGRFTISRDNAKNSLYLQMNSLRAEDTALYYCAKGSGAHYDIMTGARLDYWGQGTLVTVSS
K-COV-901_1C1_LC	DIQMTQSPSSLSASVGDRVTITCRASQSITNYLNWYQQKPGKVPKLLIYAASILQSGVPSRFSGSGSGTDFTLTISSLQPEDFATYYCQQSYSTPWTFGQGTKVEIK
